# Supplementary material for: Protocol for a longitudinal, prospective cohort study investigating the biology of uterine fibroids and endometriosis, and patients’ quality of life: the FENOX study
Source: BMJ Open. 2020 Mar 4;10(3):e032220. doi: 10.1136/bmjopen-2019-032220 (PMC7059531; doi:10.1136/bmjopen-2019-032220)
Supplement: Author's manuscript [file bmjopen-2019-032220.draft_revisions.pdf]

BMJ Open

BMJ Open is committed to open peer review. As part of this commitment we make the peer review history of every article we publish publicly available.

When an article is published we post the peer reviewers' comments and the authors' responses online. We also post the versions of the paper that were used during peer review. These are the versions that the peer review comments apply to.

The versions of the paper that follow are the versions that were submitted during the peer review process. They are not the versions of record or the final published versions. They should not be cited or distributed as the published version of this manuscript.

BMJ Open is an open access journal and the full, final, typeset and author-corrected version of record of the manuscript is available on our site with no access controls, subscription charges or pay-per-view fees (http://bmjopen.bmj.com).

If you have any questions on BMJ Open's open peer review process please email info.bmjopen@bmj.com

FENOX: Fibroids and Endometriosis Study Oxford – A Study into the Biology of Uterine Fibroids and Endometriosis.

Journal:	*BMJ Open*
Manuscript ID	bmjopen-2019-032220
Article Type:	Protocol
Date Submitted by the Author:	08-Jun-2019
Complete List of Authors:	Tapmeier, Thomas; University of Oxford, Nuffield Department of Women's & Reproductive Health<br>Nazri, Hannah; University of Oxford, Nuffield Department of Women's & Reproductive Health<br>Subramaniam, Kavita; University of Oxford, Nuffield Department of Women's & Reproductive Health<br>Manek, Sanjiv; Oxford University Hospitals NHS Foundation Trust, Department of Cellular Pathology<br>Garbutt, Kurtis; University of Oxford, Nuffield Department of Women's & Reproductive Health<br>Flint, Emma; University of Oxford, Nuffield Department of Women's & Reproductive Health<br>Cheuk, Cecilia; University of Oxford, Nuffield Department of Women's & Reproductive Health<br>Hubbard, Carol; University of Oxford, Nuffield Department of Women's & Reproductive Health<br>Barrett, Kelly; University of Oxford, Nuffield Department of Women's & Reproductive Health<br>Shepherd, Emily; University of Oxford, Nuffield Department of Women's & Reproductive Health<br>Zondervan, Krina; University of Oxford, Nuffield Department of Women's & Reproductive Health; Wellcome Trust Centre for Human Genetics<br>Becker, Christian; University of Oxford, Nuffield Department of Women's & Reproductive Health
Keywords:	GYNAECOLOGY, Reproductive medicine < GYNAECOLOGY, Subfertility < GYNAECOLOGY, REPRODUCTIVE MEDICINE, SURGERY

SCHOLARONE™
Manuscripts

I, the Submitting Author has the right to grant and does grant on behalf of all authors of the Work (as defined in the below author licence), an exclusive licence and/or a non-exclusive licence for contributions from authors who are: i) UK Crown employees; ii) where BMJ has agreed a CC-BY licence shall apply, and/or iii) in accordance with the terms applicable for US Federal Government officers or employees acting as part of their official duties; on a worldwide, perpetual, irrevocable, royalty-free basis to BMJ Publishing Group Ltd ("BMJ") its licensees and where the relevant Journal is co-owned by BMJ to the co-owners of the Journal, to publish the Work in this journal and any other BMJ products and to exploit all rights, as set out in our licence.

The Submitting Author accepts and understands that any supply made under these terms is made by BMJ to the Submitting Author unless you are acting as an employee on behalf of your employer or a postgraduate student of an affiliated institution which is paying any applicable article publishing charge ("APC") for Open Access articles. Where the Submitting Author wishes to make the Work available on an Open Access basis (and intends to pay the relevant APC), the terms of reuse of such Open Access shall be governed by a Creative Commons licence – details of these licences and which Creative Commons licence will apply to this Work are set out in our licence referred to above.

Other than as permitted in any relevant BMJ Author's Self Archiving Policies, I confirm this Work has not been accepted for publication elsewhere, is not being considered for publication elsewhere and does not duplicate material already published. I confirm all authors consent to publication of this Work and authorise the granting of this licence.

For peer review only

Title: **FENOX: Fibroids and Endometriosis Study Oxford – A Study into the Biology of Uterine Fibroids and Endometriosis.**

Authors: Tapmeier TT[1], Nazri HM[1], Subramaniam KS[1], Manek S[2], Garbutt K[1], Flint EJ[1], Cheuk C[1], Hubbard C[1], Barrett K[1], Shepherd E[1], Zondervan KT[1,3], Becker CM[1]

[1]Endometriosis CaRe Centre, Nuffield Department of Women's & Reproductive Health, University of Oxford, Oxford, UK
[2]Department of Cellular Pathology, Oxford University Hospitals, Oxford, UK
[3]Wellcome Centre for Human Genetics, University of Oxford, Oxford

For peer review only

ABSTRACT

Introduction

Millions of women suffer from the consequences of endometriosis and uterine fibroids, with fibroids the cause for over 50% of hysterectomies in the US, and direct costs for their treatment estimated at between 4 and 9 billion USD. Endometriosis commonly affects millions of women worldwide predominantly during reproductive age, with severe menstrual and non-menstrual pain and subfertility the main symptom. Due to the 'unhappy triad' of endometriosis – lack of awareness, lack of clinically relevant biomarkers and the unspecific nature of symptoms – women wait on average for 6–9 years before the definitive endometriosis diagnosis is made. Treatment options for both conditions are not satisfactory at the moment, especially with a view to preserving fertility for the women and families affected. In the FENOX study, we combine the investigation of fibroids and endometriosis, and plan to collect high quality tissue samples and medical data of participants over a time frame of 5 years after surgical intervention.

Methods and analysis

Biological samples such as blood, saliva, urine, fat, peritoneal fluid and – if found – endometrial tissue or fibroids as well as detailed clinical and intraoperative data will be collected from women participating in the study after informed consent. We plan to recruit up to 1200 participants per disease arm (i.e. endometriosis and uterine fibroids) over 5 years. Participants will fill in detailed and validated questionnaires on their medical history and quality of life, with follow-ups for 5 years. We will analyse the biological samples using state-of-the-art molecular biology methods and correlate the findings with the medical records and questionnaire data.

Ethics and dissemination

Approval for this study has been granted by the South Central - Oxford B Research Ethics Committee (REC No. 17/SC/0664), by the Health Research Authority (HRA), and by the Oxford University Hospitals NHS Foundation Trust. The findings will be published in high-ranking journals in the field and presented at national and international conferences.

Trial registration number

ISRCTN13560263; https://doi.org/10.1186/ISRCTN13560263

For peer review only

For peer review only - http://bmjopen.bmj.com/site/about/guidelines.xhtml

ARTICLE SUMMARY

Strengths and limitations of this study

- FENOX combines the study of endometriosis and uterine fibroids to identify the underlying mechanisms of both conditions.
- The study comprises biological samples as well as comprehensive phenotypic data.
- WERF EPHect criteria are applied to ensure the best possible standardisation.
- The longitudinal aspect is an important feature of FENOX but this depends on uptake and compliance by participants.

INTRODUCTION

Millions of women suffer from the consequences of endometriosis [1] and uterine fibroids [2,3]. These include pelvic and abdominal pain, abnormal uterine bleeding, infertility and miscarriages [4–7]. As such, these conditions not only affect women and their families in their everyday lives, but also have been shown to have an enormous socioeconomic impact for society in general: In the United States, fibroids are cited to be the cause for over 50% of hysterectomies [8], and direct costs for their treatment is estimated between 4 and 9 billion USD [9].

Clinically relevant, non-invasive diagnostic tests including biomarkers or imaging techniques do not exist for many forms of endometriosis [10–12] resulting in an average delay in diagnosis of 8-12 years. Current treatment options are associated with significant side effects and risks and include hormonal suppression/modification, surgical removal or, in the case of fibroids, embolization and MRI guided focussed ultrasound (MRgFUS).

Therefore, there exists a significant unmet clinical need to better understand the underlying mechanisms of these conditions, which will enable us to develop more specific diagnostic tests and will eventually lead to individualised treatment, with fewer side effects and better efficacy. To achieve this goal, it is essential to collect prospective high quality, standardised clinical and intra-operative data and corresponding biological samples. Our group has been at the forefront of the development of standard operating procedures and questionnaires for endometriosis as part of the World Endometriosis Research Foundation's (WERF) Endometriosis Phenome and Biobanking Harmonisation Project (EPHect) [13–16], and we are planning to establish similar standards in uterine fibroid research.

In the FENOX (Fibroids and Endometriosis in Oxford) study, we aim to improve our understanding of the underlying mechanisms of endometriosis and uterine fibroids and their associated symptoms by means of longitudinal observation and laboratory analyses. To achieve this, samples and clinical data

For peer review only - http://bmjopen.bmj.com/site/about/guidelines.xhtml

will be collected from women undergoing surgery. These samples will be used in state-of-the-art biomedical assays (see 'Assays') to improve our understanding of the underlying biology of these symptoms in women with endometriosis and/or fibroids, which will lead to a better understanding of the conditions, stratification of patient groups and tailored therapies, and the development of novel drug targets and biomarkers for diagnosis and treatment.

Objectives

Primary objective

- To identify the underlying mechanisms of endometriosis and uterine fibroids and their associated symptoms to improve the outcome of affected women.

Secondary objectives

- To identify novel biomarkers of endometriosis.
- To identify clinical subgroups of endometriosis and uterine fibroids.
- To understand the genetics underlying these conditions and explore the relevant downstream molecular pathways.
- To investigate the relation between the presence of fibroids and the symptoms, e.g. abnormal uterine bleeding.
- To identify novel drug targets.
- To develop models of disease progression and prediction.
- To investigate conditions or symptoms associated with endometriosis and/or uterine fibroids, including: symptoms and characteristics of the female reproductive system (characteristics of menstrual bleeding, fertility, infertility, pregnancy outcomes), pelvic as well as non-pelvic pain conditions, metabolic phenotypes (polycystic ovarian syndrome (PCOS), obesity and fat distribution), cardiovascular conditions and symptoms, neuroangiogenesis and related neurological symptoms, immunological disorders, and cancers.

Outcomes

Primary outcome

We will used questionnaire data, medical records and sample analysis to investigate the genetic and molecular basis of the pathogenesis and symptoms of endometriosis and uterine fibroids. At the end of the recruiting period, i.e. from December 2022 onwards, the collected data and samples will be analysed and compared between endometriosis/fibroid cases, and non-affected controls.

For peer review only - http://bmjopen.bmj.com/site/about/guidelines.xhtml

Secondary outcomes

Prospective standardised questionnaires and samples will be collected according to EPHect (Endometriosis Phenome and Biobanking Harmonisation Project) standards. The correlation of data and endometriosis status will allow us to define novel biomarkers of the disease.

Clinical notes and questionnaires in combination with sample data will be used to define clinical subgroups of patients.

The molecular and genetic findings will be compared against public databases of disease-relevant molecular pathways, and *in vitro* experiments will be carried out to test hypothetical connections between the genetics and manifestation of disease.

The blood vessels and endothelial cells will be compared between tissue from women presenting with fibroids and those without.

The detailed comparison between tissue from women with fibroids and those without will yield differences in terms of proteins expressed; these can then be tested as targets using known or new drugs.

As data accumulate and genetic mechanisms become clear, hypotheses will be formed as to the likely progression of disease. These will be tested against the reports from the follow-up questionnaires.

We will use questionnaire data, medical records and sample analysis to investigate the genetic and molecular basis of the pathogenesis and symptoms of conditions or symptoms associated with endometriosis and/or uterine fibroids.

METHODS AND ANALYSIS

Study design

FENOX is a prospective study that aims to improve our understanding of the underlying mechanisms of endometriosis and uterine fibroids and their associated symptoms by means of longitudinal observation and laboratory analyses. Biological samples such as blood, saliva, urine, fat, peritoneal fluid and – if found - endometrial tissue or fibroids as well as detailed clinical and intraoperative data will be collected from women of reproductive age with and without endometriosis- and fibroid-associated symptoms, such as pain, abnormal uterine bleeding and infertility. Women undergoing surgery for these conditions, and women undergoing surgery for unrelated gynaecological conditions as part of their normal clinical management will be asked to participate. All women attending clinics receive a letter informing them of ongoing research, and eligible women will be identified initially by research nurses or clinical staff during clinic visits. Once a woman has expressed an interest in participating in this study, they will be consented by a member of the research team (see Fig.1).

Blood, saliva and urine will be taken prior to surgery. Tissue and peritoneal fluid (where applicable) will be taken at the time of the scheduled surgery.

In order to determine the effect of the surgical removal of the fibroids on the local tissue, it is necessary to take an additional endometrial biopsy after the planned surgical intervention. This sample will be timed to synch with the same time point in the menstrual cycle that the original

For peer review only - http://bmjopen.bmj.com/site/about/guidelines.xhtml

sample was taken, and thus will give us a unique insight into the biology of the conditions. During this visit, blood and urine samples will be taken again also. Women can opt in or out of the additional clinic visit where these samples would be taken.

Women will be asked to complete questionnaires appropriate to their condition on paper, online or into their electronic handheld devices (health, pain, medication and, initially, ethnicity) at different time points. There will be a lengthy questionnaire at baseline and then shorter versions post-operatively at 6-8 weeks, 6 months, 12 months and thereafter yearly for a total of five years after surgical intervention.

Samples

A. Blood samples (up to 50 ml, venepuncture), urine (micturition) and saliva (spit) will be taken prior to surgery.
B. During surgery, tissue samples will be taken as specified below.
C. In women opting in, an additional endometrial biopsy will be taken during a follow-up visit at least three months after surgery. This can be done in an outpatient setting, and the taking of an endometrial biopsy in this setting using an endometrial sampling device (e.g. pipelle or curette) is an established technique. A blood and urine sample will be taken again also.

Participants

In each of the disease arms (endometriosis or fibroids), we plan to recruit up to 1200 women of reproductive age (18 years until menopause) who are planned to undergo surgery. 800 of these will have the condition of interest, and 400 will be having surgery for other reasons and act as controls. In addition, we will include fibroid and uterine tissue samples collected as excess tissue (Oxford Radcliffe Biobank, REC Ref 09/H0606/5+5), currently approximately 70 samples.

Inclusion criteria

- The participant is willing and able to give informed consent for participation in the study.
- The participant is female and aged 18 years or above (before menopause).
- Women undergoing planned surgery (including hysterectomy) for endometriosis- and/or fibroid associated symptoms such as abdominal pain, abnormal uterine bleeding, or for unrelated gynaecological conditions (e.g. fertility investigation or for laparoscopic tubal sterilisation).

Exclusion criteria

The participant may not enter the study if ANY of the following apply:

- Women who are pregnant.
- Women who are unable to read, or to understand written or spoken English.
- History of cancer/ diagnosis of current cancer.

For peer review only - http://bmjopen.bmj.com/site/about/guidelines.xhtml

Participant enrolment

Recruitment

After general information through a generic letter with information about ongoing research which every patient will receive prior to her outpatient appoint, eligible women will be identified initially by the research nurses or clinical team during clinic visits. The study research nurses will then contact those women interested in participating in the study.

Screening and Eligibility Assessment

Women attending clinic appointments for endometriosis- and fibroid-associated symptoms such as pain, abnormal uterine bleeding, and infertility will be asked to participate by clinical staff or by the authorised study research nurses. Women undergoing surgery for these conditions, and women undergoing surgery as part of their normal clinical management (e.g. laparoscopic tubal ligation or hysterectomy; they would be the control patients) are eligible to participate in the study.

Informed Consent

Prior to giving consent, and usually during their pre-operative assessment visit, women will be given the relevant patient information sheet and consent form to read. Written consent will be received by a trained member of the research team.

Written versions, with verbal explanations, of the patient information sheet and the consent forms will be presented to the participants detailing the exact nature of the study; what it will involve for the participant; the implications and constraints of the protocol; the known side effects and any risks involved in taking part. It will be clearly stated that the participant is free to withdraw from the study at any time for any reason without prejudice to future care, without affecting their legal rights, and with no obligation to give the reason for withdrawal.

The participant will be allowed as much time as wished to consider the information, and the opportunity to question the Investigator, their GP or other independent parties to decide whether they will participate in the study.

Written informed consent will then be obtained by means of the participant's dated signature and the dated signature of the person who presented and obtained the informed consent. The person who obtained the consent will be suitably qualified and experienced, and have been authorised to do so by the Chief Investigator. A copy of the signed informed consent and the patient information sheet will be given to the participant. The original signed form will be retained at the study site. The consent form for this study allows for the participant declining consent for any procedure that she is not comfortable with, while remaining eligible as a participant of the study. E.g. if a participant did not want a uterine biopsy used in the study, she would not initial the corresponding box on the consent form and insert 'No' instead.

For peer review only

For peer review only - http://bmjopen.bmj.com/site/about/guidelines.xhtml

Study settings

Baseline Assessments

Consented participants will be asked by the research team to complete a baseline questionnaire before their surgery as appropriate to their condition. This may be sent to them e.g. via mail or e-mail before surgery or given to them in paper form in the clinic. Alternatively, participants have the option to complete an online version of the questionnaire or they may fill in the questionnaire on their hand-held device. Each participant will receive a unique login for the online questionnaires.

The questionnaire data will be withheld from the research team until written informed consent is obtained, and destroyed if this is not granted.

All participants will be sent further questionnaires at different time points (approximately 6-8 weeks, 6 months, 12 months and then yearly for 5 years after surgery). Participants may be reminded (twice, maximally) to return completed questionnaires via mail, email, phone or text or similar.

On the day of surgery, they will be asked to provide a mid-stream urine and a saliva sample. In addition, blood will be collected by peripheral venepuncture. The procedures will be explained to the women again and they will be given the opportunity to ask questions. Assessment of the presence and extent of disease will be performed by the operating surgeon.

Samples that may be taken at time of surgical procedures are as follows:

1. *Laparoscopy for suspected endometriosis:*

Endometrial biopsy, peritoneal fluid aspiration, fat tissue biopsy, endometriotic tissue if present, peritoneal biopsy.

2. *Laparoscopy for uterine fibroids:*

Endometrial biopsy, peritoneal fluid aspiration, fat tissue biopsy, endometriotic tissue (if present), peritoneal biopsy, fibroid tissue, myometrial biopsy.

3. *Laparotomy for uterine fibroids:*

Endometrial biopsy, peritoneal fluid aspiration, fat tissue biopsy, endometriotic tissue if present, peritoneal biopsy, fibroid tissue, myometrial biopsy.

4. *Trans-cervical resection of uterine fibroids (TCRF):*

Endometrial biopsy, fibroid tissue, myometrial biopsy

For peer review only - http://bmjopen.bmj.com/site/about/guidelines.xhtml

5. Laparoscopy for tubal sterilisation (Controls):

Endometrial biopsy, peritoneal fluid aspiration, fat tissue biopsy, peritoneal biopsy, myometrial biopsy

6. Hysterectomy:

Whole uterus as excess tissue from women with fibroids as well as from controls without fibroids, who undergo surgery for other indications (such as heavy menstrual bleeding, or pain), peritoneal fluid aspiration, fat tissue biopsy and peritoneal biopsy.

Women undergoing hysterectomy for benign causes such as abnormal uterine bleeding or pain will be asked to donate part of their uterus for research. Hysterectomy specimens are excess tissue and would be discarded otherwise.

During surgery, the surgeon will record digital photographs of the inside of the abdomen and/or uterine cavity as part of routine clinical care, which will also be stored on a secure server identified by the participant's study ID. Intraoperative findings will be recorded by the surgeon and anonymised data collected.

Subsequent Visits

Unless participants underwent a hysterectomy, all included women will be asked to contact the study team when the next menstrual period after the procedure started, in order to calculate the length of that cycle. This is important in order to account for the changes that occur in the uterus during the menstrual cycle, and to enable us to distinguish between the effects of the cycle and the disease.

One subsequent visit will be made by participants who consent to this. They will have another endometrial sample taken at least three months after the surgical intervention in an outpatient setting. The taking of an endometrial biopsy in this setting using an endometrial sampling device (e.g. pipelle or curette) is an established technique, takes approximately 30 minutes, and there is only a minimal risk of bleeding. In addition, blood and urine samples will be taken also. Pregnant women will not be eligible for the subsequent visits.

All women will be contacted by a member of the medical or study team and asked to fill in further questionnaires at different follow-up time points (approximately 6-8 weeks, 6 months, 12 months and yearly thereafter for a total of 5 years). Reminders will be sent twice, maximally. Women will also be asked if they can be contacted in the future for any further studies approved by an ethics committee.

Sample Handling

For peer review only - http://bmjopen.bmj.com/site/about/guidelines.xhtml

Samples will be obtained by the interventions listed below. Only the study team will have access. Biological samples will be stored at -20°C or at -80°C for use in current and future studies until exhausted. Disease and control samples will be stored under the same conditions. . For the purposes of this study, samples may be analysed at Oxford, or they may be transferred to a third party/study collaborator, including industrial partners, for analysis at their facility. If participants agree, samples will be moved to a Research Tissue Bank at the end of the study, or stored and used in future ethically approved studies.They would be made available in anonymised form.

To investigate the relationship between uterine fibroids and symptoms such as abnormal uterine bleeding and pain, we aim to collect endometrial and myometrial samples alongside the fibroids themselves, to be able to detect the effects of fibroids on their surroundings.

We also intend to use the endometrium and endometriotic samples, one of the blood samples and fat samples to look for genetic factors and molecular pathways that can lead to endometriosis or uterine fibroids. The samples for this analysis will also be anonymised so that we do not know specifically which patient they came from. However, all samples are identifiable with printed label and location detail, participant ID, sample type and colour-coded cap.

Blood:

50 mL. These are divided into (at least) EDTA- (2 x 9 mL) and heparin-treated samples (6 mL, both anti-coagulation), serum (SST, 5 mL) and a plain blood sample of 5 mL. The different vials are colour-coded and frozen at -80°C.

Urine:

20 mL. Half of the sample will be used to test for glucose by specific gravity assay, the other half will be stored for the study. One aliquot of 5 mL is frozen directly at -80°C; 1 mL is centrifuged at 300 g, and 5 aliquots a 200 µL of cell-free supernatant are frozen at -80°C.

Saliva:

A spit sample of approximately 1 mL is taken on ice. One aliquot of 200 µL is frozen at -80°C, the rest is centrifuged at 300 g and 2 x 200 µL of cell-free supernatant are frozen at -80°C. One aliquot of 50 µL of cell-free supernatant is combined with 200 µL of RNA-preserving buffer (RNA later, Qiagen, Germany) and then frozen at -80°C.

Peritoneal fluid:

During surgery, the peritoneal fluid will be collected on ice. Depending on the volume (up to 15 mL), an aliquot will be centrifuged at 300 g, and the pellet (cells) stored at -80°C for further analysis. The cell-free supernatant will also be stored at -80°C.

For peer review only - http://bmjopen.bmj.com/site/about/guidelines.xhtml

324 *Endometrium (e.g. pipelle or curette), endometrial lesions (peritoneum), abdominal fat, myometrium,*
325 *fibroid tissue:*
326 All tissue will be collected on ice and divided for storage at -80°C and – after fixing in
327 paraformaldehyde and ethanol – at room temperature. Parts of fresh tissues will be used for culturing
328 experiments, in order to test compounds, drugs or similar agents on primary cells.

329 *Hysterectomy:*
330 In agreement with the local pathologist, whole uteri will be taken on ice and used for perfusion
331 experiments within 24 hours before being transferred to pathology. Tissue samples of myometrium,
332 endometrium, fibroid and fibroid-associated vasculature (if present) will be taken and stored at -80°C
333 and – after fixing in paraformaldehyde and ethanol – at room temperature as the other tissue
334 samples above.

335 *Assays*

336 *RNA analysis*
337 RNA from each sample will be isolated by standard methods. Gene expression studies will be carried
338 out between cases and controls (e.g. endometriosis vs non-endometriosis patients, or fibroid bearing
339 women vs women without fibroids) using quantitative real-time PCR assays, whole RNA sequencing
340 methods and RNA microarrays.

341 *Protein analysis*
342 Proteins will be extracted from tissue samples using standard methods. The expression and amount
343 of proteins will be analysed by immunoblotting for specific proteins of interest, and by proteomics
344 methods using the MALDI/SELDI platform. Tissue sections will be used in standard
345 immunohistochemistry to detect the expression of markers of interest *in situ*.

346 *Cells*
347 Fresh tissue will be dissociated into single cell suspensions. From these, the diverse cell types (e.g.
348 endothelial cells) will be grown in incubators *in vitro* in order to study differences in cell behaviour
349 between cases and controls, and to test compounds and drugs. Cells will be analysed by microscopy,
350 flow cytometry and immunocytochemistry methods.
351 Similarly, cells isolated from peritoneal fluid or blood will be analysed using these methods.

352 *Secretome analysis (perfusion)*
353 Whole uteri with and without fibroids will be perfused with a suitable buffer for up to 8 hours. The
354 perfusate will be analysed by proteomics methods (see above) to detect factors secreted by the
355 fibroids.

For peer review only - http://bmjopen.bmj.com/site/about/guidelines.xhtml
For peer review only

Microscopy

Tissue blocks (up to 5 cubic millimetres in size) from perfused uteri will be stained with antibodies against markers of blood vessels and fibroids, and leakiness, and be recorded in a confocal microscope in order to render a three-dimensional image of the blood vessels *in situ*. The detailed study of these will allow us to determine whether there is a significant difference in the architecture of blood vessels in uteri with fibroids compared to those from uteri without fibroids.

Discontinuation/Withdrawal of Participants from Study

Each participant has the right to withdraw from the study at any time. In addition, the Investigator may discontinue a participant from the study at any time if the Investigator considers it necessary for any reason including:

- Pregnancy
- Ineligibility (either arising during the study or retrospectively, having been missed at screening)
- Withdrawal of Consent
- Loss to follow up
- Loss of mental capacity

Withdrawal from the study: At the point the participant withdraws from the study, we will ask for consent to retain samples and data collected up to that point.

Withdrawn participants will not be replaced.

The reason for withdrawal will be recorded in the CRF.

Definition of End of Study

The end of study is six months after the locking of the study database, to allow for completion of data analysis.

Patient and Public Involvement

FENOX was built on experience and feedback we received from patients and research nurses during a previous study (ENDOX[17]). In addition, the research objectives were set in accordance with research priorities identified through the James Lind Alliance Priority Setting Partnership (PSP) for endometriosis, in which we participate[18]. The James Lind Alliance brings patients, carers and clinicians together in Priority Setting Partnerships (PSPs) to identify and prioritise the Top 10 unanswered questions or evidence uncertainties that they agree are the most important.

INTERVENTIONS

Non-clinical Interventions

For peer review only - http://bmjopen.bmj.com/site/about/guidelines.xhtml

Questionnaires:

As appropriate for their study arm – endometriosis or uterine fibroids – participants will complete specific questionnaires about general health, pain sensitivity, medication and menstrual history before their surgery. Additionally, those women with endometriosis will be asked to complete the Endometriosis Health Profile (EHP-30) Questionnaire, while the questionnaire for women with fibroids contains a section on their quality of life (UFS-QoL[19]). Currently, the clinical questionnaires are completed by participants on paper; in the future, the aim is that the questionnaires can be completed via a handheld device or via the internet directly onto a secure server. For this, women will receive a pre-trial study number and login information. The follow-up questionnaires ask about symptoms and changes in menstrual history as relevant. The control groups would be given the same questionnaires as the women with the respective condition, and asked to omit questions that are not applicable to them.

Medical records:

We will obtain clinical data (menstrual cycle phase, medication, pain and menstrual bleeding status, photos from surgery) from the patients' medical records.

Clinical Interventions

Venepuncture:

Taken by an appropriately trained member of the clinical or research staff.

Collection of other bodily fluid sample:

Urine and saliva samples donated by the patient and sample prepared and analysed by a member of the investigative team.

Tissue collection:

Tissue/fluid (e.g. fibroids if present) will be collected as part of routine surgical management apart from:

Laparoscopy:

Peritoneal fluid will be aspirated, biopsies from endometrium (e.g. pipelle or curette), abdominal fat tissue, myometrium, and peritoneum (excision) will be taken during surgery.

Additional Risk:

Minor bleeding, uterine perforation (<1%), additional length of surgery by 5 min.

Myomectomy/hysterectomy:

Endometrial, myometrial and/or fibroid tissue biopsies will be taken during surgery. Hysterectomy samples will be used in structural analysis assays *ex vivo* in close discussion with the clinical pathologists.

Additional Risk: Minor bleeding, uterine perforation (<1%), additional length of surgery by 5 min.

For peer review only - http://bmjopen.bmj.com/site/about/guidelines.xhtml

Trans-cervical resection of fibroids:

Endometrial and myometrial biopsies will be taken during surgery.

Additional Risk: Minor bleeding, uterine perforation (<1%), additional length of surgery by 5 min.

Additionally:

In women opting in, an additional endometrial biopsy will be taken during a follow-up visit n an outpatient setting. The biopsy of the endometrium is a simple, routine procedure and takes about 30 minutes.

Additional risk: Minor bleeding, uterine perforation (<1%), short period of discomfort.

Women will be asked to consent to the use of samples and clinical data collected as part of this research and in future research. Women, if they consent, will potentially be contacted for future studies approved by an ethics committee.

Adverse events

For this study, it is conceivable that additional procedures may result in bleeding. However, if this resulted in a scenario mentioned below, it would constitute an SAE and needed to be reported to the sponsor.

A serious adverse event is any untoward medical occurrence that:

- results in death
- is life-threatening
- requires inpatient hospitalisation or prolongation of existing hospitalisation
- results in persistent or significant disability/incapacity
- consists of a congenital anomaly or birth defect

Other 'important medical events' may also be considered serious if they jeopardise the participant or require an intervention to prevent one of the above consequences.

NOTE: The term "life-threatening" in the definition of "serious" refers to an event in which the participant was at risk of death at the time of the event; it does not refer to an event which hypothetically might have caused death if it were more severe.

A serious adverse event (SAE) occurring to a participant should be reported to the REC that gave a favourable opinion of the study where in the opinion of the Chief Investigator the event was 'related' (resulted from administration of any of the research procedures) and 'unexpected' in relation to those procedures. Reports of related and unexpected SAEs should be submitted within 15 working days of the Chief Investigator becoming aware of the event, using the HRA report of serious adverse event form (see HRA website).

For peer review only - http://bmjopen.bmj.com/site/about/guidelines.xhtml

Description of Statistical Methods

As this is a prospective sample and data collection study, there is no randomisation of patients as all women will undergo surgery as part of their routine clinical management. We routinely use SPSS, Graph Pad Prism, STATA and R for analysis, and employ T-tests, ANOVA, correlation coefficient analysis and similar methods. Due to the exploratory nature of this study, various additional statistical techniques may also be used to fully explore the relationships in the data but all methods will be fully documented.

The Number of Participants

It is now recognised that both endometriosis and uterine fibroids are very heterogeneous conditions. Our previous studies[20,21] and systematic reviews[22,23] have clearly identified a lack of sufficiently powered studies. Multiple large-scale research collaborations are currently in place investigating different aspects of endometriosis[24], and we plan similar efforts for uterine fibroids. Therefore, large patient numbers are needed.

The Endometriosis CaRe Centre at Oxford is the UK's largest endometriosis centre. Similarly, as a tertiary referral centre, we see many women with fibroid–associated symptoms. As a result, we have the unique opportunity to collect large amounts of data and samples, which is essential to produce clinically meaningful outputs. Given our current recruitment rate (endometriosis: 100/year, uterine fibroids, 200/year) we estimate the recruitment of approximately 2 × 1200 women over the course of the study (800 endometriosis patients + 400 non-endometriotic controls, 800 fibroid patients + 400 non-fibroid controls). Fibroids already collected as excess tissue under the Oxford Radcliffe Biobank (ORB, REC Ref 09/H0606/5+5) will also be included in this study, currently approximately 70 samples.

Analysis of Outcome Measures

All samples excluding those from patients who withdraw consent will be included in the analysis of outcome measures.

Laboratory data will be analysed using assay-specific software packages employing univariate and multivariate pattern recognition methods (e.g. principal component analysis, partial least squares, stochastic neighbour embedding algorithms) between sample groups. Correlation with questionnaire data will allow us to validate prospective markers of disease. In addition, we will use laboratory data to predict disease severity (revised American Fertility Score), quality of life (EHP-30), pain measures and improvement of symptoms as per follow-up questionnaires. For the multivariate predictive methods, a test set of approximately 30% of each treatment group will be selected at random. This may be selected in a stratified method and exclude patients that have particularly extreme values. Patients not included in the test set will make up the training set. Models will then be built on the training set and assessed for predictability on the test set.

The analysis will be performed on the whole data set. However, if some influential differences are seen, then e.g. the women with endometriosis will be matched to corresponding women without endometriosis, or women with fibroids to women without fibroids, and the analysis based on these matched pairs.

For peer review only - http://bmjopen.bmj.com/site/about/guidelines.xhtml

DATA MANAGEMENT

Access to Data

Direct access will be granted to authorised representatives from the Sponsor and host institution for monitoring and/or audit of the study to ensure compliance with regulations.

Data Recording and Record Keeping

Each participant will receive a unique study number, which will then be used throughout the study. A study master sheet linking patient identifiable data (name, DOB, hospital and NHS numbers) with the unique study number will be kept and password protected on the University of Oxford's High Compliance server with authorised access and in a file separate from the main study file. Hard copy study documents will be kept in a locked room at each participating centre. Research data will therefore be using non-identifiable data, and all records will be identified only by this study number. All study data will be entered on a desktop computer into a program such as Microsoft EXCEL or Sapphire (Labvantage) using password protection. The participants will be identified by study number in any database. The name and any other identifying details will NOT be included in any electronic file of study data.

Where participants consent, coded genetic data and limited relevant details including, age, gender, information about body habitus, biochemistry etc. can also be made available to collaborators and to the National Institute for Health Research (NIHR) Bioresource (http://bioresource.nihr.ac.uk/), a panel of thousands of volunteers, who are willing to be approached to participate in research studies investigating the links between genes, the environment, health and disease.

QUALITY ASSURANCE PROCEDURES

The study may be monitored, or audited in accordance with the current approved protocol, GCP, relevant regulations and standard operating procedures.

ETHICAL AND REGULATORY CONSIDERATIONS

Declaration of Helsinki

This study will be conducted in accordance with the principles of the Declaration of Helsinki.

Guidelines for Good Clinical Practice

The Investigator will ensure that this study is conducted in accordance with relevant regulations and with Good Clinical Practice.

Approvals

The protocol, informed consent form, participant information sheet and any proposed advertising material will be submitted to an appropriate Research Ethics Committee (REC), and HRA for written approval.

The Investigator will submit and, where necessary, obtain approval from the above parties for all substantial amendments to the original approved documents.

For peer review only - http://bmjopen.bmj.com/site/about/guidelines.xhtml

534 **Reporting**

535 The CI shall submit once a year throughout the study, or on request, an Annual Progress report to the REC Committee, HRA (where required) host organisation and Sponsor. In addition, an End of Study notification and final report will be submitted to the same parties.

538 **Participant Confidentiality**

539 The study staff will ensure that the participants' anonymity is maintained. The participants will be identified only by a participant ID number on all study documents and any electronic database, with the exception of the CRF, where participant initials may be added. All documents will be stored securely and only accessible by study staff and authorised personnel. The study will comply with the Data Protection Act, which requires data to be anonymised as soon as it is practical to do so.

544 **Expenses and Benefits**

545 There will be no payments made to study participants.

546 **Other Ethical Considerations**

547 Participants unable to consent for themselves will not be included in the study.

548 Patients under clinical management for infertility will be approached in a most sensitive manner by our experienced and well-trained team. It is unlikely that our genetic analysis of the participants will reveal anything relevant beyond their normal clinical care so we do not plan to report any such findings to them or their GPs.

553 **DISCUSSION**

554 This study has been designed to address both uterine fibroids and endometriosis as conditions that both affect the female reproductive system and pose similar problems with regards to pain treatment, fertility and quality of life. By combining the patient collectives into one study, we hope to make use of synergies between the investigations of the two conditions, in addition to the apparent comorbidity between endometriosis patients and those with uterine fibroids [25,26]. Uniquely, the study protocol allows for the sampling of endometrium on a follow-up visit, which will allow for the assessment of the local, molecular effects of treatment within the same participant.

561 FENOX has been designed with the EPHect principles [13–16] in mind to ensure standardisation and reproducibility, and thus should deliver high-quality datasets that will be useful and comparable between centres. We are currently expanding the collection of samples to sites outside of Oxford, with a view to make FENOX a multi-centre study within the UK eventually.

565 *Author affiliations*

566 [1]Endometriosis CaRe Centre, Nuffield Department of Women's & Reproductive Health, University of Oxford, Oxford, UK

568 [2]Department of Cellular Pathology, Oxford University Hospitals, Oxford, UK

569 [3]Wellcome Centre for Human Genetics, University of Oxford, Oxford

For peer review only - http://bmjopen.bmj.com/site/about/guidelines.xhtml

Acknowledgements

We thank all women who participated in FENOX and its predecessor, ENDOX. We acknowledge the indispensable help and expertise of Fiona Goddard, NDWRH, and Karen Melham, CTRG, University of Oxford, in preparing the study protocol.

Contributors

TTT wrote the study protocol with KTZ and CMB. CH, KB and ES consented participants and collected samples. HMN, KSS and TTT processed samples. KG, EJF and CC documented samples and designed electronic questionnaires. SM determined menstrual cycle stages by histology. KTZ and CMB conceived the study.

Funding

TTT received funding from the Nuffield Benefaction for Medicine and the Wellcome Institutional Strategic Support Fund (ISSF, ref no. 5258). HMN received funding from the Oxfordshire Health Services Research Committee. KG was supported by a grant from the National Institutes of Health USA (R01HD094842). Funding for this study has been obtained from the Nuffield Department of Women's & Reproductive Health under the Oxford/Bayer-Alliance for Women's Health.

Competing interests

CMB and KTZ received research grants from Bayer Healthcare, Volition RX, MDNA Life Sciences and Roche Diagnostics. The study is funded by the Nuffield Department of Women's & Reproductive Health.

Patient consent

See above, *informed consent*

Ethics approval

Approval has been granted by the South Central - Oxford B Research Ethics Committee (REC No. 17/SC/0664), by the Health Research Authority (HRA), and by the Oxford University Hospitals NHS Foundation Trust.

Provenance and peer review

The FENOX protocol was based upon the previous ENDOX study (REC reference 09/H0604/58). It was originally approved in February 2018, with amendments approved in April 2019.

Open access

The study protocol will be published under a Creative Commons licence.

For peer review only - http://bmjopen.bmj.com/site/about/guidelines.xhtml

REFERENCES

1 Berkley KJ, Rapkin AJ, Papka RE. The pains of endometriosis. *Science* 2005;**308**:1587–9. doi:10.1126/science.1111445

2 Peddada SD, Laughlin SK, Miner K, *et al.* Growth of uterine leiomyomata among premenopausal black and white women. *Proc Natl Acad Sci* 2008;**105**:19887–92. doi:10.1073/pnas.0808188105

3 Buttram VC, Reiter RC. Uterine leiomyomata: etiology, symptomatology, and management. *Fertil Steril* 1981;**36**:433–45.http://www.ncbi.nlm.nih.gov/pubmed/7026295

4 Burney RO, Giudice LC. Pathogenesis and pathophysiology of endometriosis. *Fertil Steril* 2012;**98**:511–9. doi:10.1016/j.fertnstert.2012.06.029

5 Giudice LC. Clinical practice. Endometriosis. *N Engl J Med* 2010;**362**:2389–98. doi:10.1056/NEJMcp1000274

6 Gupta S, Jose J, Manyonda I. Clinical presentation of fibroids. *Best Pract Res Clin Obstet Gynaecol* 2008;**22**:615–26. doi:10.1016/j.bpobgyn.2008.01.008

7 Longo DL, Bulun SE. Uterine Fibroids. *N Engl J Med* 2013;**369**:1344–55. doi:10.1056/NEJMra1209993

8 Aarts JWM, Nieboer TE, Johnson N, *et al.* Surgical approach to hysterectomy for benign gynaecological disease. *Cochrane Database Syst Rev* Published Online First: 2015. doi:10.1002/14651858.CD003677.pub5

9 Cardozo ER, Clark AD, Banks NK, *et al.* The estimated annual cost of uterine leiomyomata in the United States. *Am J Obstet Gynecol* 2012;**206**:211.e1-211.e9. doi:10.1016/j.ajog.2011.12.002

10 Gupta D, Hull ML, Fraser I, *et al.* Endometrial biomarkers for the non-invasive diagnosis of endometriosis. *Cochrane database Syst Rev* 2016;**4**:CD012165. doi:10.1002/14651858.CD012165

11 Nisenblat V, Prentice L, Bossuyt PMM, *et al.* Combination of the non-invasive tests for the diagnosis of endometriosis. *Cochrane database Syst Rev* 2016;**7**:CD012281. doi:10.1002/14651858.CD012281

12 Nisenblat V, Bossuyt PMM, Shaikh R, *et al.* Blood biomarkers for the non-invasive diagnosis of endometriosis. *Cochrane database Syst Rev* 2016;:CD012179. doi:10.1002/14651858.CD012179

13 Becker CM, Laufer MR, Stratton P, *et al.* World Endometriosis Research Foundation Endometriosis Phenome and Biobanking Harmonisation Project: I. Surgical phenotype data collection in endometriosis research. *Fertil Steril* 2014;**102**:1213–22. doi:10.1016/j.fertnstert.2014.07.709

For peer review only - http://bmjopen.bmj.com/site/about/guidelines.xhtml

14 Vitonis AF, Vincent K, Rahmioglu N, *et al.* World Endometriosis Research Foundation Endometriosis Phenome and Biobanking Harmonization Project: II. Clinical and covariate phenotype data collection in endometriosis research. *Fertil Steril* 2014;**102**:1223–32. doi:10.1016/j.fertnstert.2014.07.1244

15 Rahmioglu N, Fassbender A, Vitonis AF, *et al.* World Endometriosis Research Foundation Endometriosis Phenome and Biobanking Harmonization Project: III. Fluid biospecimen collection, processing, and storage in endometriosis research. *Fertil Steril* 2014;**102**:1233–43. doi:10.1016/j.fertnstert.2014.07.1208

16 Fassbender A, Rahmioglu N, Vitonis AF, *et al.* World Endometriosis Research Foundation Endometriosis Phenome and Biobanking Harmonisation Project: IV. Tissue collection, processing, and storage in endometriosis research. *Fertil Steril* 2014;**102**:1244–53. doi:10.1016/j.fertnstert.2014.07.1209

17 Rahmioglu N, Drong AW, Lockstone H, *et al.* Variability of genome-wide DNA methylation and mRNA expression profiles in reproductive and endocrine disease related tissues. *Epigenetics* 2017;**12**:897–908. doi:10.1080/15592294.2017.1367475

18 Horne AW, Saunders PTK, Abokhrais IM, *et al.* Top ten endometriosis research priorities in the UK and Ireland. *Lancet (London, England)* 2017;**389**:2191–2. doi:10.1016/S0140-6736(17)31344-2

19 Spies JB, Coyne K, Guaou Guaou N, *et al.* The UFS-QOL, a new disease-specific symptom and health-related quality of life questionnaire for leiomyomata. *Obstet Gynecol* 2002;**99**:290–300.http://www.ncbi.nlm.nih.gov/pubmed/11814511

20 Rahmioglu N, Nyholt DR, Morris AP, *et al.* Genetic variants underlying risk of endometriosis: insights from meta-analysis of eight genome-wide association and replication datasets. *Hum Reprod Update* 2014;**20**:702–16. doi:10.1093/humupd/dmu015

21 Fung JN, Rogers PAW, Montgomery GW. Identifying the Biological Basis of GWAS Hits for Endometriosis. *Biol Reprod* 2015;**92**:87–87. doi:10.1095/biolreprod.114.126458

22 May KE, Villar J, Kirtley S, *et al.* Endometrial alterations in endometriosis: a systematic review of putative biomarkers. *Hum Reprod Update* 2011;**17**:637–53. doi:10.1093/humupd/dmr013

23 May KE, Conduit-Hulbert SA, Villar J, *et al.* Peripheral biomarkers of endometriosis: a systematic review. *Hum Reprod Update* 2010;**16**:651–74. doi:10.1093/humupd/dmq009

24 Sapkota Y, Steinthorsdottir V, Morris AP, *et al.* Meta-analysis identifies five novel loci associated with endometriosis highlighting key genes involved in hormone metabolism. *Nat Commun* 2017;**8**:15539. doi:10.1038/ncomms15539

25 Choi EJ, Cho SB, Lee SR, *et al.* Comorbidity of gynecological and non-gynecological diseases with adenomyosis and endometriosis. *Obstet Gynecol Sci* 2017;**60**:579–86. doi:10.5468/ogs.2017.60.6.579

26 Huang JQ, Lathi RB, Lemyre M, *et al.* Coexistence of endometriosis in women with symptomatic leiomyomas. *Fertil Steril* 2010;**94**:720–3. doi:10.1016/j.fertnstert.2009.03.052

For peer review only - http://bmjopen.bmj.com/site/about/guidelines.xhtml

676

For peer review only - http://bmjopen.bmj.com/site/about/guidelines.xhtml

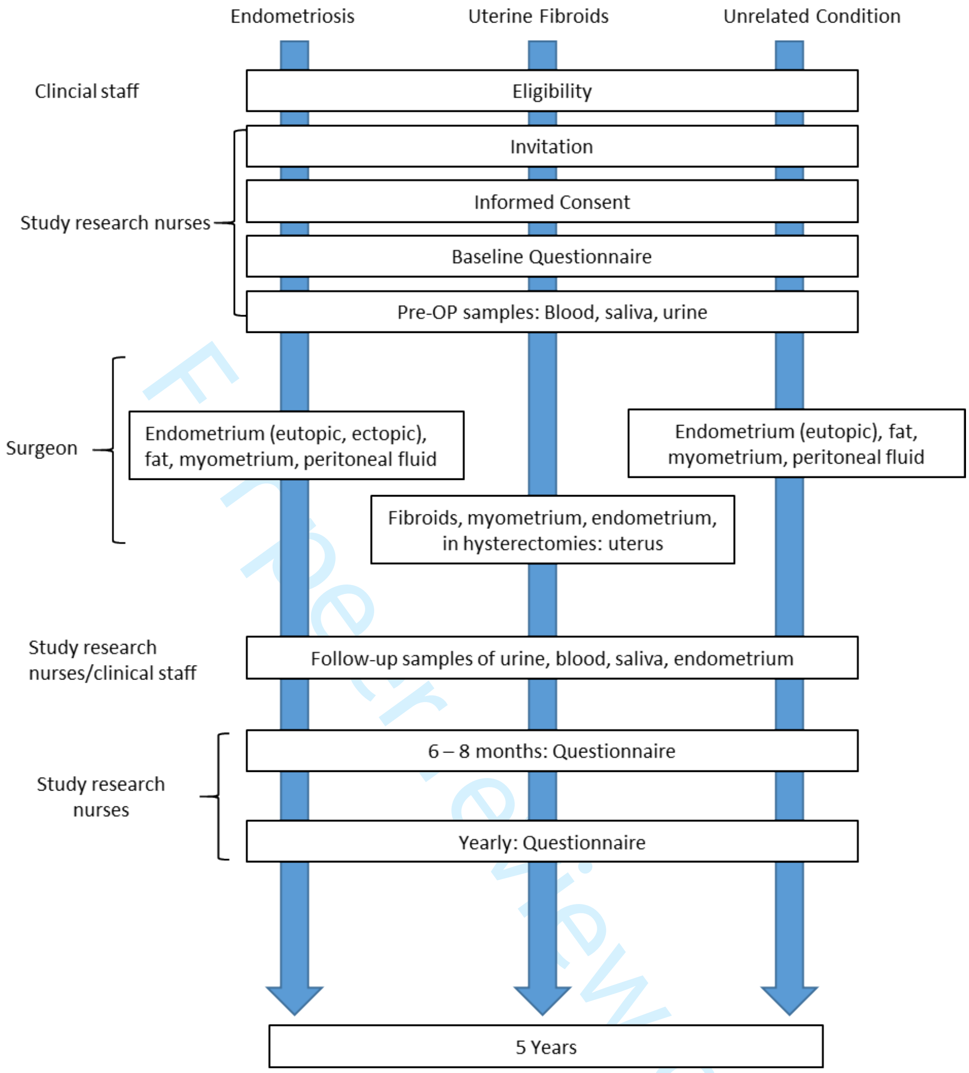

Figure 1: Flowchart of data and tissue sampling.

For peer review only

BMJ Open

A protocol for a longitudinal, prospective cohort study investigating the biology of uterine fibroids and endometriosis, and patients' quality of life – the FENOX study.

Journal:	*BMJ Open*
Manuscript ID	bmjopen-2019-032220.R1
Article Type:	Protocol
Date Submitted by the Author:	09-Dec-2019
Complete List of Authors:	Tapmeier, Thomas; University of Oxford, Nuffield Department of Women's & Reproductive Health<br>Nazri, Hannah; University of Oxford, Nuffield Department of Women's & Reproductive Health<br>Subramaniam, Kavita; University of Oxford, Nuffield Department of Women's & Reproductive Health<br>Manek, Sanjiv; Oxford University Hospitals NHS Foundation Trust, Department of Cellular Pathology<br>Garbutt, Kurtis; University of Oxford, Nuffield Department of Women's & Reproductive Health<br>Flint, Emma; University of Oxford, Nuffield Department of Women's & Reproductive Health<br>Cheuk, Cecilia; University of Oxford, Nuffield Department of Women's & Reproductive Health<br>Hubbard, Carol; University of Oxford, Nuffield Department of Women's & Reproductive Health<br>Barrett, Kelly; University of Oxford, Nuffield Department of Women's & Reproductive Health<br>Shepherd, Emily; University of Oxford, Nuffield Department of Women's & Reproductive Health<br>Zondervan, Krina; University of Oxford, Nuffield Department of Women's & Reproductive Health; Wellcome Trust Centre for Human Genetics<br>Becker, Christian; University of Oxford, Nuffield Department of Women's & Reproductive Health
**Primary Subject Heading**:	Obstetrics and gynaecology
Secondary Subject Heading:	Genetics and genomics, Reproductive medicine, Epidemiology
Keywords:	GYNAECOLOGY, Reproductive medicine < GYNAECOLOGY, Subfertility < GYNAECOLOGY, REPRODUCTIVE MEDICINE, SURGERY

SCHOLARONE™
Manuscripts

I, the Submitting Author has the right to grant and does grant on behalf of all authors of the Work (as defined in the below author licence), an exclusive licence and/or a non-exclusive licence for contributions from authors who are: i) UK Crown employees; ii) where BMJ has agreed a CC-BY licence shall apply, and/or iii) in accordance with the terms applicable for US Federal Government officers or employees acting as part of their official duties; on a worldwide, perpetual, irrevocable, royalty-free basis to BMJ Publishing Group Ltd ("BMJ") its licensees and where the relevant Journal is co-owned by BMJ to the co-owners of the Journal, to publish the Work in this journal and any other BMJ products and to exploit all rights, as set out in our licence.

The Submitting Author accepts and understands that any supply made under these terms is made by BMJ to the Submitting Author unless you are acting as an employee on behalf of your employer or a postgraduate student of an affiliated institution which is paying any applicable article publishing charge ("APC") for Open Access articles. Where the Submitting Author wishes to make the Work available on an Open Access basis (and intends to pay the relevant APC), the terms of reuse of such Open Access shall be governed by a Creative Commons licence – details of these licences and which Creative Commons licence will apply to this Work are set out in our licence referred to above.

Other than as permitted in any relevant BMJ Author's Self Archiving Policies, I confirm this Work has not been accepted for publication elsewhere, is not being considered for publication elsewhere and does not duplicate material already published. I confirm all authors consent to publication of this Work and authorise the granting of this licence.

For peer review only

Journal: **BMJ Open – Study Protocol**

Title: **A protocol for a longitudinal, prospective cohort study investigating the biology of uterine fibroids and endometriosis, and patients' quality of life – the FENOX study.**

Authors: Tapmeier TT[1], Nazri HM[1], Subramaniam KS[1], Manek S[2], Garbutt K[1], Flint EJ[1], Cheuk C[1], Hubbard C[1], Barrett K[1], Shepherd E[1], Zondervan KT[1,3], Becker CM[1]

[1]Endometriosis CaRe Centre, Nuffield Department of Women's & Reproductive Health, University of Oxford, Oxford, UK

[2]Department of Cellular Pathology, Oxford University Hospitals, Oxford, UK

[3]Wellcome Centre for Human Genetics, University of Oxford, Oxford

Corresponding authors: thomas.tapmeier@wrh.ox.ac.uk; christian.becker@wrh.ox.ac.uk

ABSTRACT

Introduction

Millions of women suffer from the consequences of endometriosis and uterine fibroids, with fibroids the cause for over 50% of hysterectomies in the US, and direct costs for their treatment estimated at between 4 and 9 billion USD. Endometriosis commonly affects millions of women worldwide predominantly during reproductive age, with severe menstrual and non-menstrual pain and subfertility the main symptoms. Due to the 'unhappy triad' of endometriosis – lack of awareness, lack of clinically relevant biomarkers and the unspecific nature of symptoms – women wait on average for 8–12 years before the definitive endometriosis diagnosis is made. Treatment options for both conditions are not satisfactory at the moment, especially with a view to preserving fertility for the women and families affected. In the FENOX study, we combine the investigation of fibroids and endometriosis, and plan to collect high quality tissue samples and medical data of participants over a time frame of 5 years after surgical intervention.

Methods and analysis

Biological samples such as blood, saliva, urine, fat, peritoneal fluid and – if found – endometrial tissue or fibroids as well as detailed clinical and intraoperative data will be collected from women undergoing surgery and participating in the study after informed consent. We plan to recruit up to 1200 participants per disease arm (i.e. endometriosis and uterine fibroids) over 5 years. Participants will fill in detailed and validated questionnaires on their medical history and quality of life, with follow-ups for 5 years. Enrolment started on 2nd April 2018, and FENOX will close on 31st March 2028. We will analyse the biological samples using state-of-the-art molecular biology methods and correlate the findings with the medical records and questionnaire data.

For peer review only

Ethics and dissemination

Approval for this study has been granted by the South Central - Oxford B Research Ethics Committee (REC No. 17/SC/0664), by the Health Research Authority (HRA), and by the Oxford University Hospitals NHS Foundation Trust. The findings will be published in high-ranking journals in the field and presented at national and international conferences.

Trial registration number

ISRCTN13560263; https://doi.org/10.1186/ISRCTN13560263

For peer review only - http://bmjopen.bmj.com/site/about/guidelines.xhtml

ARTICLE SUMMARY

Strengths and limitations of this study

- FENOX combines the study of endometriosis and uterine fibroids to identify the underlying mechanisms of both conditions.
- The study comprises biological samples as well as comprehensive phenotypic data.
- WERF EPHect criteria are applied to ensure the best possible standardisation.
- The longitudinal aspect is an important feature of FENOX but this depends on uptake and compliance by participants.
- The control group comprises of women undergoing surgery for gynaecological indications other than endometriosis or uterine fibroids; thus, they are not completely healthy controls. This is a limitation of this study. However, we cannot ethically source tissue samples from healthy individuals.

For peer review only

INTRODUCTION

Millions of women suffer from the consequences of endometriosis [1,2] and uterine fibroids [3,4]. These include pelvic and abdominal pain, abnormal uterine bleeding, infertility and miscarriages [5–8]. As such, these conditions not only affect women and their families in their everyday lives, but also have been shown to have an enormous socioeconomic impact for society in general: In the United States, fibroids are cited to be the cause for over 50% of hysterectomies [9], and direct costs for their treatment is estimated between 4 and 9 billion USD [10].

Clinically relevant, non-invasive diagnostic tests including biomarkers or imaging techniques do not exist for many forms of endometriosis [11–13] resulting in an average delay in diagnosis of 8-12 years. Current treatment options are associated with significant side effects and risks and include hormonal suppression/modification, surgical removal or, in the case of fibroids, embolization and MRI guided focussed ultrasound (MRgFUS).

Therefore, there exists a significant unmet clinical need to better understand the underlying mechanisms of these conditions, which will enable us to develop more specific diagnostic tests and will eventually lead to individualised treatment, with fewer side effects and better efficacy. To achieve this goal, it is essential to collect prospective high quality, standardised clinical and intra-operative data and corresponding biological samples. Our group has been at the forefront of the development of standard operating procedures and questionnaires for endometriosis as part of the World Endometriosis Research Foundation's (WERF) Endometriosis Phenome and Biobanking Harmonisation Project (EPHect) [14–17], and we are planning to establish similar standards in uterine fibroid research.

In the FENOX (Fibroids and Endometriosis in Oxford) study, we aim to improve our understanding of the underlying mechanisms of endometriosis and uterine fibroids and their associated symptoms by means of longitudinal observation and laboratory analyses. To achieve this, samples and clinical data

For peer review only - http://bmjopen.bmj.com/site/about/guidelines.xhtml

will be collected from women undergoing surgery. These samples will be used in state-of-the-art biomedical assays (see 'Assays') to improve our understanding of the underlying biology of these symptoms in women with endometriosis and/or fibroids, which will lead to a better understanding of the conditions, stratification of patient groups and tailored therapies, and the development of novel drug targets and biomarkers for diagnosis and treatment.

Objectives

Primary objective

- To identify the underlying mechanisms of endometriosis and uterine fibroids and their associated symptoms to improve the outcome of affected women.

Secondary objectives

- To identify novel biomarkers of endometriosis.
- To identify clinical subgroups of endometriosis and uterine fibroids.
- To understand the genetics underlying these conditions and explore the relevant downstream molecular pathways.
- To investigate the relation between the presence of fibroids and the symptoms, e.g. abnormal uterine bleeding.
- To identify novel drug targets.
- To develop models of disease progression and prediction.
- To investigate conditions or symptoms associated with endometriosis and/or uterine fibroids, including: symptoms and characteristics of the female reproductive system (characteristics of menstrual bleeding, fertility, infertility, pregnancy outcomes), pelvic as well as non-pelvic pain conditions, metabolic phenotypes (polycystic ovarian syndrome (PCOS), obesity and fat distribution), cardiovascular conditions and symptoms, neuroangiogenesis and related neurological symptoms, immunological disorders, and cancers.

Outcomes

Primary outcome

We will used questionnaire data, medical records and sample analysis to investigate the genetic and molecular basis of the pathogenesis and symptoms of endometriosis and uterine fibroids. At the end of the recruiting period, i.e. from December 2022 onwards, the collected data and samples will be analysed and compared between endometriosis/fibroid cases, and non-affected controls.

Secondary outcomes

Prospective standardised questionnaires and samples will be collected according to EPHect (Endometriosis Phenome and Biobanking Harmonisation Project) standards. The correlation of cellular,

For peer review only - http://bmjopen.bmj.com/site/about/guidelines.xhtml

molecular and genetic data and endometriosis status will allow us to define novel biomarkers of the disease.

Clinical notes and questionnaires in combination with sample data will be used to define clinical subgroups of patients.

The molecular and genetic findings will be compared against public databases of disease-relevant molecular pathways, and *in vitro* experiments will be carried out to test hypothetical connections between the genetics and manifestation of disease.

The blood vessels and endothelial cells will be compared between tissue from women presenting with fibroids and those without.

The detailed comparison between tissue from women with fibroids and those without will yield differences in terms of proteins expressed; these can then be tested as targets using known or new drugs.

As data accumulate and genetic mechanisms become clear, hypotheses will be formed as to the likely progression of disease. These will be tested against the reports from the follow-up questionnaires.

We will use questionnaire data, medical records and sample analysis to investigate the genetic and molecular basis of the pathogenesis and symptoms of conditions or symptoms associated with endometriosis and/or uterine fibroids.

METHODS AND ANALYSIS

Study design

FENOX is a prospective study that aims to improve our understanding of the underlying mechanisms of endometriosis and uterine fibroids and their associated symptoms by means of longitudinal observation and laboratory analyses. Biological samples such as blood, saliva, urine, fat, peritoneal fluid and – if found - endometriosis tissue or fibroids as well as detailed clinical and intraoperative data will be collected from women of reproductive age with and without endometriosis- and fibroid-associated symptoms, such as pain, abnormal uterine bleeding and infertility. Women undergoing surgery for these conditions, and women undergoing surgery for unrelated gynaecological conditions as part of their normal clinical management will be asked to participate (Fig. 1). An incidental diagnosis of endometriosis or uterine fibroids will lead to the patients' inclusion into the relevant case groups. All women attending clinics receive a letter informing them of ongoing research, and eligible women will be identified initially by research nurses or clinical staff during clinic visits. Once a woman has expressed an interest in participating in this study, they will be consented by a member of the research team (flowchart, Fig. 2).

Blood, saliva and urine will be taken prior to surgery. Tissue and peritoneal fluid (where applicable) will be taken at the time of the scheduled surgery.

In order to determine the effect of the surgical removal of the fibroids on the local tissue, it is necessary to take an additional endometrial biopsy after the planned surgical intervention. This sample will be timed to synch with the same time point in the menstrual cycle that the original sample was taken, and

For peer review only - http://bmjopen.bmj.com/site/about/guidelines.xhtml

thus will give us a unique insight into the biology of the conditions. During this visit, blood and urine samples will be taken again also. Women can opt in or out of the additional clinic visit where these samples would be taken.

Women will be asked to complete questionnaires on paper, online or into their electronic handheld devices (health, pain, medication and, initially, ethnicity) at different time points. There will be a lengthy questionnaire at baseline before surgery (taking an estimated 45 minutes to complete), and shorter versions (taking up to 30 minutes to complete) post-operatively at 6-8 weeks, 6 months, 12 months and thereafter yearly for a total of five years after surgical intervention.

Samples

A. Blood samples (up to 50 ml, venepuncture), urine (micturition) and saliva (spit) will be taken prior to surgery.
B. During surgery, tissue samples will be taken as specified below.
C. In women opting in, an additional endometrial biopsy will be taken during a follow-up visit at least three months after surgery. This can be done in an outpatient setting, and the taking of an endometrial biopsy in this setting using an endometrial sampling device (e.g. pipelle or curette) is an established technique. A blood and urine sample will be taken again also.

Participants

In each of the disease arms (endometriosis or fibroids), we plan to recruit up to 1200 women of reproductive age (18 years until menopause) who are planned to undergo surgery. 800 of these will have the condition of interest, and 400 will be having surgery for other reasons and act as controls. In addition, we will include fibroid and uterine tissue samples collected as excess tissue (Oxford Radcliffe Biobank, REC Ref 09/H0606/5+5), currently approximately 70 samples.

Inclusion criteria

- The participant is willing and able to give informed consent for participation in the study.
- The participant is female and aged 18 years or above (before menopause).
- Women undergoing planned surgery (including hysterectomy) for endometriosis- and/or fibroid associated symptoms such as abdominal pain, abnormal uterine bleeding, or for unrelated gynaecological conditions (e.g. fertility investigation or for laparoscopic tubal sterilisation).

Exclusion criteria

The participant may not enter the study if ANY of the following apply:

- Women who are pregnant.
- Women who are unable to read, or to understand written or spoken English.
- History of cancer/ diagnosis of current cancer.

For peer review only - http://bmjopen.bmj.com/site/about/guidelines.xhtml

Participant enrolment

Recruitment

After general information through a generic letter with information about ongoing research, which every patient will receive prior to her outpatient appointment, eligible women will be identified initially by the research nurses or clinical team during clinic visits. The study research nurses will then contact those women interested in participating in the study.

Screening and Eligibility Assessment

Women attending clinic appointments for endometriosis- and fibroid-associated symptoms such as pain, abnormal uterine bleeding, and infertility will be asked to participate by clinical staff or by the authorised study research nurses. Women undergoing surgery for these conditions, and women undergoing surgery as part of their normal clinical management (e.g. laparoscopic tubal ligation or hysterectomy; they would be the control patients) are eligible to participate in the study.

Informed Consent

Prior to giving consent, and usually during their pre-operative assessment visit, women will be given the relevant patient information sheet and consent form to read. Written consent will be received by a trained member of the research team.

Written versions, with verbal explanations, of the patient information sheet and the consent forms will be presented to the participants detailing the exact nature of the study; what it will involve for the participant; the implications and constraints of the protocol; the known side effects and any risks involved in taking part. It will be clearly stated that the participant is free to withdraw from the study at any time for any reason without prejudice to future care, without affecting their legal rights, and with no obligation to give the reason for withdrawal.

The participant will be allowed as much time as wished to consider the information, and the opportunity to question the Investigator, their GP or other independent parties to decide whether they will participate in the study.

Written informed consent will then be obtained by means of the participant's dated signature and the dated signature of the person who presented and obtained the informed consent. The person who obtained the consent will be suitably qualified and experienced, and will have been authorised to do so by the Chief Investigator. A copy of the signed informed consent and the patient information sheet will be given to the participant. The original signed form will be retained at the study site. The consent form for this study allows for the participant declining consent for any procedure that she is not comfortable with, while remaining eligible as a participant of the study. E.g. if a participant did not want a uterine biopsy used in the study, she would not initial the corresponding box on the consent form and insert 'No' instead.

For peer review only - http://bmjopen.bmj.com/site/about/guidelines.xhtml

Study settings

Baseline Assessments

Consented participants will be asked by the research team to complete a baseline questionnaire before their surgery. This may be sent to them e.g. via mail or e-mail before surgery or given to them in paper form in the clinic. Alternatively, participants have the option to complete an online version of the questionnaire or they may fill in the questionnaire on their hand-held device. Each participant will receive a unique login for the online questionnaires.

The questionnaire data will be withheld from the research team until written informed consent is obtained and will be destroyed if this is not granted.

All participants will be sent further questionnaires at different time points (approximately 6-8 weeks, 6 months, 12 months and then yearly for 5 years after surgery). Participants may be reminded (twice, maximally) to return completed questionnaires via mail, email, phone or text or similar.

On the day of surgery, they will be asked to provide a mid-stream urine and a saliva sample. In addition, blood will be collected by peripheral venepuncture. The procedures will be explained to the women again and they will be given the opportunity to ask questions. Assessment of the presence and extent of disease will be performed by the operating surgeon.

Samples that may be taken at time of surgical procedures are as follows:

1. *Laparoscopy for suspected endometriosis:*

Endometrial biopsy, peritoneal fluid aspiration, fat tissue biopsy, endometriotic tissue if present, peritoneal biopsy.

2. *Laparoscopy for uterine fibroids:*

Endometrial biopsy, peritoneal fluid aspiration, fat tissue biopsy, endometriotic tissue (if present), peritoneal biopsy, fibroid tissue, myometrial biopsy.

3. *Laparotomy for uterine fibroids:*

Endometrial biopsy, peritoneal fluid aspiration, fat tissue biopsy, endometriotic tissue if present, peritoneal biopsy, fibroid tissue, myometrial biopsy.

4. *Trans-cervical resection of uterine fibroids (TCRF):*

Endometrial biopsy, fibroid tissue, myometrial biopsy

For peer review only - http://bmjopen.bmj.com/site/about/guidelines.xhtml

5. *Laparoscopy for tubal sterilisation (Controls):*

Endometrial biopsy, peritoneal fluid aspiration, fat tissue biopsy, peritoneal biopsy, myometrial biopsy

6. *Hysterectomy:*

Whole uterus as excess tissue from women with fibroids as well as from controls without fibroids, who undergo surgery for other indications (such as heavy menstrual bleeding, or pain), peritoneal fluid aspiration, fat tissue biopsy and peritoneal biopsy.

Women undergoing hysterectomy for benign causes such as abnormal uterine bleeding or pain will be asked to donate part of their uterus for research. Hysterectomy specimens are excess tissue and would be discarded otherwise.

During surgery, the surgeon will record digital photographs of the inside of the abdomen and/or uterine cavity as part of routine clinical care, which will also be stored on a secure server identified by the participant's study ID. Intraoperative findings will be recorded by the surgeon and anonymised data collected.

Subsequent Visits

Unless participants underwent a hysterectomy, all included women will be asked to contact the study team when the next menstrual period after the procedure started. Together with the last menstrual period (LMP) date given at the time of the procedure, this date will be used to calculate the length of the cycle. This is important in order to account for the changes that occur in the uterus during the menstrual cycle, and to enable us to distinguish between the effects of the cycle and the disease.

One subsequent visit will be made by participants treated for fibroids who consent to this. They will have another endometrial sample taken at least three months after the surgical intervention in an outpatient setting. The taking of an endometrial biopsy in this setting using an endometrial sampling device (e.g. pipelle or curette) is an established technique, takes approximately 30 minutes, and there is only a minimal risk of bleeding. In addition, blood and urine samples will be taken also. Pregnant women will not be eligible for the subsequent visits.

All women will be contacted by a member of the medical or study team and asked to fill in further questionnaires at different follow-up time points (approximately 6-8 weeks, 6 months, 12 months and yearly thereafter for a total of 5 years). Reminders will be sent twice, maximally. Women will also be asked if they can be contacted in the future for any further studies approved by an ethics committee.

Sample Handling

For peer review only - http://bmjopen.bmj.com/site/about/guidelines.xhtml

Samples will be obtained according to WERF/EPHect guidelines[14–17] by the interventions listed below. Only the study team will have access. Biological samples will be stored at -20°C or at -80°C for use in current and future studies until exhausted. Disease and control samples will be stored under the same conditions. For the purposes of this study, samples may be analysed at Oxford, or they may be transferred to a third party/study collaborator, including industrial partners, for analysis at their facility. If participants agree, samples will be moved to a Research Tissue Bank at the end of the study or stored and used in future ethically approved studies. They would be made available in anonymised form.

To investigate the relationship between uterine fibroids and symptoms such as abnormal uterine bleeding and pain, we aim to collect endometrial and myometrial samples alongside the fibroids themselves, to be able to detect the effects of fibroids on their surroundings.

We also intend to use the endometrium and endometriotic samples, one of the blood samples and fat samples to look for genetic factors and molecular pathways that can lead to endometriosis or uterine fibroids. The samples for this analysis will also be anonymised so that we do not know specifically which patient they came from. However, all samples are identifiable with printed label and location detail, participant ID, sample type and colour-coded cap.

Blood:

50 mL. These are divided into (at least) EDTA- (2 x 9 mL) and heparin-treated samples (2 x 6 mL, both anti-coagulation), serum (2 x SST, 5 mL) and two plain blood samples of 5 mL. The different vials are colour-coded and frozen at -80°C.

Urine:

20 mL. Half of the sample will be used to test for glucose by specific gravity assay, the other half will be stored for the study. One aliquot of 5 mL is frozen directly at -80°C; 1 mL is centrifuged at 300 g, and 5 aliquots a 200 µL of cell-free supernatant are frozen at -80°C.

Saliva:

A spit sample of approximately 1 mL is taken on ice. One aliquot of 200 µL is frozen at -80°C, the rest is centrifuged at 300 g and 2 x 200 µL of cell-free supernatant are frozen at -80°C. One aliquot of 50 µL of cell-free supernatant is combined with 200 µL of RNA-preserving buffer (RNA later, Qiagen, Germany) and then frozen at -80°C.

Peritoneal fluid:

During surgery, the peritoneal fluid will be collected by the surgeon using a syringe or through mechanical suction on ice. Depending on the volume (up to 15 mL), an aliquot will be centrifuged at 300 g, and the pellet (cells) stored at -80°C for further analysis. The cell-free supernatant will also be stored at -80°C.

For peer review only - http://bmjopen.bmj.com/site/about/guidelines.xhtml

Endometrium (e.g. pipelle or curette), endometrial lesions (peritoneum), abdominal fat, myometrium, fibroid tissue:

All tissue will be collected on ice and divided for storage at -80°C and – after fixing in paraformaldehyde and ethanol – at room temperature. Parts of fresh tissues will be used for culturing experiments, in order to test compounds, drugs or similar agents on primary cells.

Hysterectomy:

In agreement with the local pathologist, whole uteri will be taken on ice and used for perfusion experiments within 24 hours before being transferred to pathology. Tissue samples of myometrium, endometrium, fibroid and fibroid-associated vasculature (if present) will be taken and stored at -80°C and – after fixing in paraformaldehyde and ethanol – at room temperature as the other tissue samples above.

Assays

RNA analysis

RNA from each sample will be isolated by standard methods. Gene expression studies will be carried out between cases and controls (e.g. endometriosis vs non-endometriosis patients, or fibroid bearing women vs women without fibroids) using quantitative real-time PCR assays, whole RNA sequencing methods and RNA microarrays.

Protein analysis

Proteins will be extracted from tissue samples using standard methods. The expression and amount of proteins will be analysed by immunoblotting for specific proteins of interest, and by proteomics methods using the MALDI/SELDI platform. Tissue sections will be used in standard immunohistochemistry to detect the expression of markers of interest *in situ*.

Cells

Fresh tissue will be dissociated into single cell suspensions. From these, the diverse cell types (e.g. endothelial cells) will be grown in incubators *in vitro* in order to study differences in cell behaviour between cases and controls, and to test compounds and drugs. Cells will be analysed by microscopy, flow cytometry and immunocytochemistry methods.

Similarly, cells isolated from peritoneal fluid or blood will be analysed using these methods.

Secretome analysis (perfusion)

Whole uteri with and without fibroids will be perfused with a suitable buffer for up to 8 hours. The perfusate will be analysed by proteomics methods (see above) to detect factors secreted by the fibroids.

For peer review only

Microscopy

Tissue blocks (up to 5 cubic millimetres in size) from perfused uteri will be stained with antibodies against markers of blood vessels and fibroids, and leakiness, and be recorded in a confocal microscope in order to render a three-dimensional image of the blood vessels *in situ*. The detailed study of these will allow us to determine whether there is a significant difference in the architecture of blood vessels in uteri with fibroids compared to those from uteri without fibroids.

Discontinuation/Withdrawal of Participants from Study

Each participant has the right to withdraw from the study at any time. In addition, the Investigator may discontinue a participant from the study at any time if the Investigator considers it necessary for any reason including:

- Pregnancy
- Ineligibility (either arising during the study or retrospectively, having been missed at screening)
- Withdrawal of Consent
- Loss to follow up
- Loss of mental capacity

Withdrawal from the study: At the point the participant withdraws from the study, we will ask for consent to retain samples and data collected up to that point.

Withdrawn participants will not be replaced.

The reason for withdrawal will be recorded in the CRF.

Definition of End of Study

The end of study is six months after the locking of the study database, to allow for completion of data analysis.

Patient and Public Involvement

FENOX was built on experience and feedback we received from patients and research nurses during a previous study (A study to identify possible biomarkers in women with Endometriosis at Oxford – ENDOX[18]). In addition, the research objectives were set in accordance with research priorities identified through the James Lind Alliance Priority Setting Partnership (PSP) for endometriosis, in which we participate[19]. The James Lind Alliance brings patients, carers and clinicians together in Priority Setting Partnerships (PSPs) to identify and prioritise the Top 10 unanswered questions or evidence uncertainties that they agree are the most important.

INTERVENTIONS

Non-clinical Interventions

For peer review only - http://bmjopen.bmj.com/site/about/guidelines.xhtml

Questionnaires:

Participants will complete specific questionnaires about their condition, general health, pain sensitivity, medication and menstrual history before their surgery. Additionally, those women with endometriosis will be asked to complete the Endometriosis Health Profile (EHP-30) Questionnaire[20], while women with fibroids complete a section on their quality of life (UFS-QoL[21]). Currently, the clinical questionnaires are completed by participants on paper; in the future, the aim is that the questionnaires can be completed via a handheld device or via the internet directly onto a secure server. For this, women will receive a pre-trial study number and login information. The follow-up questionnaires ask about symptoms and changes in menstrual history as relevant. The control groups would be given the same questionnaires as the women with the respective condition and asked to omit questions not applicable to them.

Medical records:

We will obtain clinical data (menstrual cycle phase, medication, pain and menstrual bleeding status, photos from surgery) from the patients' medical records.

Clinical Interventions

Venepuncture:

Taken by an appropriately trained member of the clinical or research staff.

Collection of other bodily fluid sample:

Urine and saliva samples donated by the patient and sample prepared and analysed by a member of the investigative team.

Tissue collection:

Tissue/fluid (e.g. fibroids if present) will be collected as part of routine surgical management apart from:

Laparoscopy:

Peritoneal fluid will be aspirated, biopsies from endometrium (e.g. pipelle or curette), abdominal fat tissue, myometrium, and peritoneum (excision) will be taken during surgery.

Additional Risk:

Minor bleeding, uterine perforation (<1%), additional length of surgery by 5 min.

Myomectomy/hysterectomy:

Endometrial, myometrial and/or fibroid tissue biopsies will be taken during surgery. Hysterectomy samples will be used in structural analysis assays *ex vivo* in close discussion with the clinical pathologists.

Additional Risk: Minor bleeding, uterine perforation (<1%), additional length of surgery by 5 min.

For peer review only

Trans-cervical resection of fibroids:

Endometrial and myometrial biopsies will be taken during surgery.

Additional Risk: Minor bleeding, uterine perforation (<1%), additional length of surgery by 5 min.

Additionally:

In women opting in, an additional endometrial biopsy will be taken during a follow-up visit n an outpatient setting. The biopsy of the endometrium is a simple, routine procedure and takes about 30 minutes.

Additional risk: Minor bleeding, uterine perforation (<1%), short period of discomfort.

Women will be asked to consent to the use of samples and clinical data collected as part of this research and in future research. Women, if they consent, will potentially be contacted for future studies approved by an ethics committee.

Adverse events

For this study, it is conceivable that additional procedures may result in bleeding. However, if this resulted in a scenario mentioned below, it would constitute an SAE and needed to be reported to the sponsor.

A serious adverse event is any untoward medical occurrence that:

- results in death
- is life-threatening
- requires inpatient hospitalisation or prolongation of existing hospitalisation
- results in persistent or significant disability/incapacity
- consists of a congenital anomaly or birth defect

Other 'important medical events' may also be considered serious if they jeopardise the participant or require an intervention to prevent one of the above consequences.

NOTE: The term "life-threatening" in the definition of "serious" refers to an event in which the participant was at risk of death at the time of the event; it does not refer to an event which hypothetically might have caused death if it were more severe.

A serious adverse event (SAE) occurring to a participant should be reported to the REC that gave a favourable opinion of the study where in the opinion of the Chief Investigator the event was 'related' (resulted from administration of any of the research procedures) and 'unexpected' in relation to those procedures. Reports of related and unexpected SAEs should be submitted within 15 working days of the Chief Investigator becoming aware of the event, using the HRA report of serious adverse event form (see HRA website).

For peer review only - http://bmjopen.bmj.com/site/about/guidelines.xhtml

Data Analysis Plan

As this is a prospective sample and data collection study, there is no randomisation of patients as all women will undergo surgery as part of their routine clinical management. As previously[18], we will use SPSS, Graph Pad Prism, STATA and R for analysis, and employ t-tests, ANOVA, correlation coefficient analysis and similar methods. We plan to use multivariate logistic regression models in comparisons of endometriosis cases with controls, and fibroid cases with controls, adjusting for confounders relevant to the hypothesis being tested. A priori confounders are likely to be age, ethnicity and menstrual cycle phase. Patients with both endometriosis and fibroids will enter into the analysis according to the research question asked; we will conduct sensitivity analyses on this comorbid group to examine to what extent they influence the results. Due to the exploratory nature of this study, various additional statistical techniques may also be used to fully explore the relationships in the data, but all methods will be fully documented.

Power Calculations

Power calculations were done in R (v3.6.1) using the ***pwr*** package. For our primary outcome we plan to correlate questionnaire and laboratory data. To detect correlations with a moderate effect size of r=0.3 at a power of 80% and 0.05 significance level, we will need 85 samples per group (***pwr.r.test***). For the detection of effect sizes of at least 0.2 between groups (e.g. endometriosis cases vs controls, with 3 cycle phases and 5 disease stages (0, stages 1 – 4) using ANOVA at 0.05 significance and 80% power, at least 32 samples per groups will be used, with a total of 480 samples for all 15 groups (***pwr.anova.test***). Multiple comparisons will be corrected for by Bonferroni's method.

The Number of Participants

It is now recognised that both endometriosis and uterine fibroids are very heterogeneous conditions. Our previous studies[22,23] and systematic reviews[24,25] have clearly identified a lack of sufficiently powered studies. Multiple large-scale research collaborations are currently in place investigating different aspects of endometriosis[26], and we plan similar efforts for uterine fibroids. Therefore, large patient numbers are needed.

The Endometriosis CaRe Centre at Oxford is the UK's largest endometriosis centre. Similarly, as a tertiary referral centre, we see many women with fibroid–associated symptoms. As a result, we have the unique opportunity to collect large amounts of data and samples, which is essential to produce clinically meaningful outputs. Given our current patient recruitment rate (endometriosis: 100/year, uterine fibroids, 200/year) we estimate an enrolment of approximately 2 × 1200 women over the course of the study (800 endometriosis patients + 400 non-endometriotic controls, 800 fibroid patients + 400 non-fibroid controls). Fibroids already collected as excess tissue under the Oxford Radcliffe Biobank (ORB, REC Ref 09/H0606/5+5) will also be included in this study, currently approximately 70 samples.

Analysis of Outcome Measures

All samples excluding those from patients who withdraw consent will be included in the analysis of outcome measures.

For peer review only - http://bmjopen.bmj.com/site/about/guidelines.xhtml

Laboratory data will be analysed using assay-specific software packages employing univariate and multivariate pattern recognition methods (e.g. principal component analysis, partial least squares, stochastic neighbour embedding algorithms) between sample groups. Correlation with questionnaire data will allow us to validate prospective markers of disease. In addition, we will use laboratory data to predict disease severity (revised American Fertility Society score[27]), quality of life (EHP-30[20]), pain measures and improvement of symptoms as per follow-up questionnaires. For the multivariate predictive methods, a test set of approximately 30% of each treatment group will be selected at random. This may be selected in a stratified method and exclude patients that have particularly extreme values (e.g. > 3 SD from the mean). Patients not included in the test set will make up the training set. Models will then be built on the training set and assessed for predictability on the test set. The final analysis will be performed on the whole data set. However, if some influential differences e.g. in BMI or comorbidities are seen, then e.g. the women with endometriosis will be matched to corresponding women without endometriosis, or women with fibroids to women without fibroids, and the analysis based on these matched pairs.

DATA MANAGEMENT

Access to Data

Direct access will be granted to authorised representatives from the Sponsor and host institution for monitoring and/or audit of the study to ensure compliance with regulations.

Data Recording and Record Keeping

Each participant will receive a unique study number, which will then be used throughout the study. A study master sheet linking patient identifiable data (name, DOB, hospital and NHS numbers) with the unique study number will be kept and password protected on the University of Oxford's High Compliance server with authorised access and in a file separate from the main study file. Hard copy study documents will be kept in a locked room at each participating centre. Research data will therefore be using non-identifiable data, and all records will be identified only by this study number. All study data will be entered on a desktop computer into a program such as Microsoft EXCEL or Sapphire (Labvantage) using password protection. The participants will be identified by study number in any database. The name and any other identifying details will NOT be included in any electronic file of study data.

Where participants consent, coded genetic data and limited relevant details including, age, gender, information about body habitus, biochemistry etc. can also be made available to collaborators and to the National Institute for Health Research (NIHR) Bioresource (http://bioresource.nihr.ac.uk/), a panel of thousands of volunteers, who are willing to be approached to participate in research studies investigating the links between genes, the environment, health and disease.

QUALITY ASSURANCE PROCEDURES

For peer review only - http://bmjopen.bmj.com/site/about/guidelines.xhtml

The study may be monitored, or audited in accordance with the current approved protocol, GCP, relevant regulations and standard operating procedures.

ETHICAL AND REGULATORY CONSIDERATIONS

Declaration of Helsinki

This study will be conducted in accordance with the principles of the Declaration of Helsinki.

Guidelines for Good Clinical Practice

The Investigator will ensure that this study is conducted in accordance with relevant regulations and with Good Clinical Practice.

Approvals

The protocol, informed consent form, participant information sheet and any proposed advertising material will be submitted to an appropriate Research Ethics Committee (REC), and HRA for written approval.

The Investigator will submit and, where necessary, obtain approval from the above parties for all substantial amendments to the original approved documents.

Reporting

The CI shall submit once a year throughout the study, or on request, an Annual Progress report to the REC Committee, HRA (where required) host organisation and Sponsor. In addition, an End of Study notification and final report will be submitted to the same parties.

Participant Confidentiality

The study staff will ensure that the participants' anonymity is maintained. The participants will be identified only by a participant ID number on all study documents and any electronic database, with the exception of the CRF, where participant initials may be added. All documents will be stored securely and only accessible by study staff and authorised personnel. The study will comply with the Data Protection Act, which requires data to be anonymised as soon as it is practical to do so.

Expenses and Benefits

There will be no payments made to study participants.

Other Ethical Considerations

Participants unable to consent for themselves will not be included in the study.

Patients under clinical management for infertility will be approached in a most sensitive manner by our experienced and well-trained team. It is unlikely that our genetic analysis of the participants will reveal anything relevant beyond their normal clinical care so we do not plan to report any such findings to them or their GPs.

ETHICS AND DISSEMINATION

For peer review only

For peer review only - http://bmjopen.bmj.com/site/about/guidelines.xhtml

Approval for this study has been granted by the South Central - Oxford B Research Ethics Committee (REC No. 17/SC/0664) on 31st January 2018, by the Health Research Authority (HRA) on 28th February 2018, and by the Oxford University Hospitals NHS Foundation Trust on 20th March 2018.

The findings will be published in high-ranking journals in the field and presented at national and international conferences.

DISCUSSION

This study has been designed to address both uterine fibroids and endometriosis as conditions that both affect the female reproductive system and pose similar problems with regards to pain treatment, fertility and quality of life. By combining the patient collectives into one study, we hope to make use of synergies between the investigations of the two conditions, in addition to the apparent comorbidity between endometriosis patients and those with uterine fibroids [28,29]. Uniquely, the study protocol allows for the sampling of endometrium on a follow-up visit, which will allow for the assessment of the local, molecular effects of treatment within the same participant.

FENOX has been designed with the EPHect principles [14–17] in mind to ensure standardisation and reproducibility, and thus should deliver high-quality datasets that will be useful and comparable between centres. We are currently expanding the collection of samples to sites outside of Oxford, with a view to make FENOX a multi-centre study within the UK eventually.

Author affiliations

[1]Endometriosis CaRe Centre, Nuffield Department of Women's & Reproductive Health, University of Oxford, Oxford, UK

[2]Department of Cellular Pathology, Oxford University Hospitals, Oxford, UK

[3]Wellcome Centre for Human Genetics, University of Oxford, Oxford

Acknowledgements

We thank all women who participated in FENOX and its predecessor, ENDOX. We acknowledge the indispensable help and expertise of Fiona Goddard, NDWRH, and Karen Melham, CTRG, University of Oxford, in preparing the study protocol.

Contributors

TTT wrote the study protocol with KTZ and CMB. CH, KB and ES consented participants and collected samples. HMN, KSS and TTT processed samples. KG, EJF and CC documented samples and designed electronic questionnaires. SM determined menstrual cycle stages by histology. KTZ and CMB conceived the study.

For peer review only

For peer review only - http://bmjopen.bmj.com/site/about/guidelines.xhtml

Funding

TTT received funding from the Nuffield Benefaction for Medicine and the Wellcome Institutional Strategic Support Fund (ISSF, ref no. 5258). HMN received funding from the Oxfordshire Health Services Research Committee. KG was supported by a grant from the National Institutes of Health USA (R01HD094842). Funding for this study has been obtained from the Nuffield Department of Women's & Reproductive Health under the Oxford/Bayer-Alliance for Women's Health.

Competing interests

CMB and KTZ received research grants from Bayer Healthcare, Volition RX, MDNA Life Sciences and Roche Diagnostics. The study is funded by the Nuffield Department of Women's & Reproductive Health.

Patient consent

See above, *informed consent*

Ethics approval

Approval has been granted by the South Central - Oxford B Research Ethics Committee (REC No. 17/SC/0664), by the Health Research Authority (HRA), and by the Oxford University Hospitals NHS Foundation Trust.

Provenance and peer review

The FENOX protocol was based upon the previous ENDOX study (REC reference 09/H0604/58). It was originally approved in January 2018, with amendments approved in April 2019.

Open access

The study protocol will be published under a Creative Commons licence.

Data sharing

The authors will make relevant anonymised patient-level data available upon reasonable request, according to the established standards in the field. Data that could compromise participant anonymity or privacy will not be shared in any way.

For peer review only

For peer review only - http://bmjopen.bmj.com/site/about/guidelines.xhtml

REFERENCES

1 Berkley KJ. The Pains of Endometriosis. *Science (80-)* 2005;**308**:1587–9. doi:10.1126/science.1111445

2 Zondervan KT, Becker CM, Koga K, *et al.* Endometriosis. *Nat Rev Dis Prim* 2018;**4**:9. doi:10.1038/s41572-018-0008-5

3 Peddada SD, Laughlin SK, Miner K, *et al.* Growth of uterine leiomyomata among premenopausal black and white women. *Proc Natl Acad Sci* 2008;**105**:19887–92. doi:10.1073/pnas.0808188105

4 Buttram VC, Reiter RC. Uterine leiomyomata: etiology, symptomatology, and management. *Fertil Steril* 1981;**36**:433–45.http://www.ncbi.nlm.nih.gov/pubmed/7026295

5 Burney RO, Giudice LC. Pathogenesis and pathophysiology of endometriosis. *Fertil Steril* 2012;**98**:511–9. doi:10.1016/j.fertnstert.2012.06.029

6 Giudice LC. Clinical practice. Endometriosis. *N Engl J Med* 2010;**362**:2389–98. doi:10.1056/NEJMcp1000274

7 Gupta S, Jose J, Manyonda I. Clinical presentation of fibroids. *Best Pract Res Clin Obstet Gynaecol* 2008;**22**:615–26. doi:10.1016/j.bpobgyn.2008.01.008

8 Longo DL, Bulun SE. Uterine Fibroids. *N Engl J Med* 2013;**369**:1344–55. doi:10.1056/NEJMra1209993

9 Aarts JWM, Nieboer TE, Johnson N, *et al.* Surgical approach to hysterectomy for benign gynaecological disease. *Cochrane Database Syst Rev* Published Online First: 2015. doi:10.1002/14651858.CD003677.pub5

10 Cardozo ER, Clark AD, Banks NK, *et al.* The estimated annual cost of uterine leiomyomata in the United States. *Am J Obstet Gynecol* 2012;**206**:211.e1-211.e9. doi:10.1016/j.ajog.2011.12.002

11 Gupta D, Hull ML, Fraser I, *et al.* Endometrial biomarkers for the non-invasive diagnosis of endometriosis. *Cochrane database Syst Rev* 2016;**4**:CD012165. doi:10.1002/14651858.CD012165

12 Nisenblat V, Prentice L, Bossuyt PM, *et al.* Combination of the non-invasive tests for the diagnosis of endometriosis. *Cochrane Database Syst Rev* 2016;**7**:CD012281. doi:10.1002/14651858.CD012281

13 Nisenblat V, Bossuyt PM, Shaikh R, *et al.* Blood biomarkers for the non-invasive diagnosis of endometriosis. *Cochrane Database Syst Rev* 2016;:CD012179. doi:10.1002/14651858.CD012179

14 Becker CM, Laufer MR, Stratton P, *et al.* World Endometriosis Research Foundation Endometriosis Phenome and Biobanking Harmonisation Project: I. Surgical phenotype data collection in endometriosis research. *Fertil Steril* 2014;**102**:1213–22. doi:10.1016/j.fertnstert.2014.07.709

15 Vitonis AF, Vincent K, Rahmioglu N, *et al.* World Endometriosis Research Foundation Endometriosis Phenome and Biobanking Harmonization Project: II. Clinical and covariate

For peer review only - http://bmjopen.bmj.com/site/about/guidelines.xhtml

phenotype data collection in endometriosis research. *Fertil Steril* 2014;**102**:1223–32. doi:10.1016/j.fertnstert.2014.07.1244

16 Rahmioglu N, Fassbender A, Vitonis AF, *et al.* World Endometriosis Research Foundation Endometriosis Phenome and Biobanking Harmonization Project: III. Fluid biospecimen collection, processing, and storage in endometriosis research. *Fertil Steril* 2014;**102**:1233–43. doi:10.1016/j.fertnstert.2014.07.1208

17 Fassbender A, Rahmioglu N, Vitonis AF, *et al.* World Endometriosis Research Foundation Endometriosis Phenome and Biobanking Harmonisation Project: IV. Tissue collection, processing, and storage in endometriosis research. *Fertil Steril* 2014;**102**:1244–53. doi:10.1016/j.fertnstert.2014.07.1209

18 Rahmioglu N, Drong AW, Lockstone H, *et al.* Variability of genome-wide DNA methylation and mRNA expression profiles in reproductive and endocrine disease related tissues. *Epigenetics* 2017;**12**:897–908. doi:10.1080/15592294.2017.1367475

19 Horne AW, Saunders PTK, Abokhrais IM, *et al.* Top ten endometriosis research priorities in the UK and Ireland. *Lancet (London, England)* 2017;**389**:2191–2. doi:10.1016/S0140-6736(17)31344-2

20 Jones G. Development of an endometriosis quality-of-life instrument: The Endometriosis Health Profile-30. *Obstet Gynecol* 2001;**98**:258–64. doi:10.1016/S0029-7844(01)01433-8

21 Spies JB, Coyne K, Guaou Guaou N, *et al.* The UFS-QOL, a new disease-specific symptom and health-related quality of life questionnaire for leiomyomata. *Obstet Gynecol* 2002;**99**:290–300.http://www.ncbi.nlm.nih.gov/pubmed/11814511

22 Rahmioglu N, Nyholt DR, Morris AP, *et al.* Genetic variants underlying risk of endometriosis: insights from meta-analysis of eight genome-wide association and replication datasets. *Hum Reprod Update* 2014;**20**:702–16. doi:10.1093/humupd/dmu015

23 Fung JN, Rogers PAW, Montgomery GW. Identifying the Biological Basis of GWAS Hits for Endometriosis. *Biol Reprod* 2015;**92**:87–87. doi:10.1095/biolreprod.114.126458

24 May KE, Villar J, Kirtley S, *et al.* Endometrial alterations in endometriosis: a systematic review of putative biomarkers. *Hum Reprod Update* 2011;**17**:637–53. doi:10.1093/humupd/dmr013

25 May KE, Conduit-Hulbert SA, Villar J, *et al.* Peripheral biomarkers of endometriosis: a systematic review. *Hum Reprod Update* 2010;**16**:651–74. doi:10.1093/humupd/dmq009

26 Sapkota Y, Steinthorsdottir V, Morris AP, *et al.* Meta-analysis identifies five novel loci associated with endometriosis highlighting key genes involved in hormone metabolism. *Nat Commun* 2017;**8**:15539. doi:10.1038/ncomms15539

27 The American Society for Reproductive Medicine. Revised American Society for Reproductive Medicine classification of endometriosis: 1996. *Fertil Steril* 1997;**67**:817–21.http://www.ncbi.nlm.nih.gov/pubmed/9130884

28 Choi EJ, Cho SB, Lee SR, *et al.* Comorbidity of gynecological and non-gynecological diseases with adenomyosis and endometriosis. *Obstet Gynecol Sci* 2017;**60**:579–86.

For peer review only - http://bmjopen.bmj.com/site/about/guidelines.xhtml

doi:10.5468/ogs.2017.60.6.579

29 Huang JQ, Lathi RB, Lemyre M, *et al.* Coexistence of endometriosis in women with symptomatic leiomyomas. *Fertil Steril* 2010;**94**:720–3. doi:10.1016/j.fertnstert.2009.03.052

For peer review only

For peer review only - http://bmjopen.bmj.com/site/about/guidelines.xhtml

FIGURE LEGENDS

Figure 1: Participant groups and procedures. The participants will be allocated into case or control groups and tissues will be sourced according to the course of clinical intervention: Participants with fibroids treated by transcervical resection (TCRF) will not undergo abdominal surgery, while abdominal surgery is necessary if the fibroids are treated by laparoscopy, open myomectomy or hysterectomy. In these cases, peritoneal fluid can be obtained in addition to the tissue samples (fibroid, myometrium, endometrium). Endometriosis patients will undergo laparoscopy. If found without endometriosis, they will be grouped with the controls, who are women undergoing surgery for other, unrelated conditions (e.g. tubular ligation).

Figure 2: Flowchart of participant data and tissue sampling. Consented participants of the different arms of the study donate pre-operative samples and fill in a baseline questionnaire. They can donate a range of tissue samples according to their condition and treatment mode (e.g. fibroids or endometriotic tissue, peritoneal fluid), and are asked to repeatedly fill in follow-up questionnaires on their condition and quality of life, so that the clinical findings can be correlated with the outcome years later.

For peer review only

For peer review only - http://bmjopen.bmj.com/site/about/guidelines.xhtml

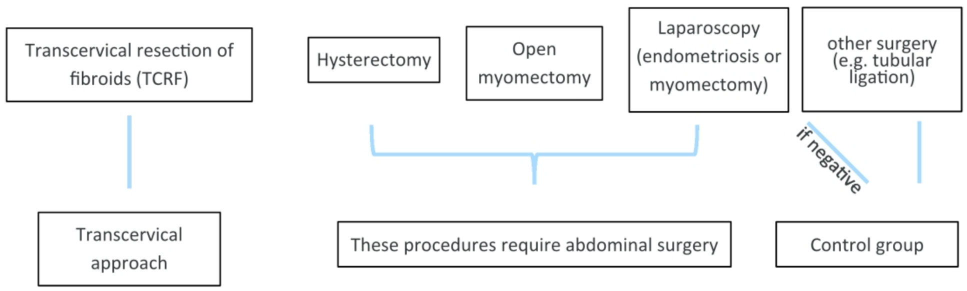

Figure 1: Participant groups and procedures. The participants will be allocated into case or control groups and tissues will be sourced according to the course of clinical intervention: Participants with fibroids treated by transcervical resection (TCRF) will not undergo abdominal surgery, while abdominal surgery is necessary if the fibroids are treated by laparoscopy, open myomectomy or hysterectomy. In these cases, peritoneal fluid can be obtained in addition to the tissue samples (fibroid, myometrium, endometrium). Endometriosis patients will undergo laparoscopy. If found without endometriosis, they will be grouped with the controls, who are women undergoing surgery for other, unrelated conditions (e.g. tubular ligation).

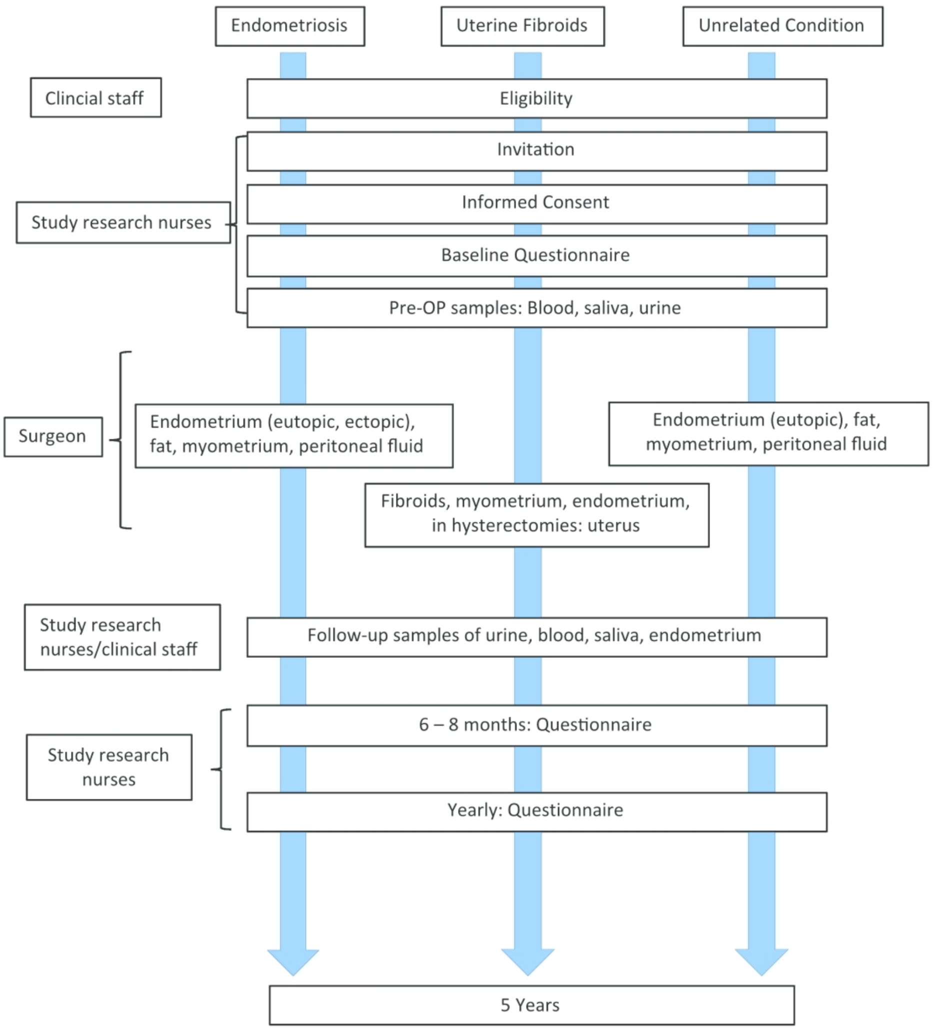

Figure 2: Flowchart of participant data and tissue sampling. Consented participants of the different arms of the study donate pre-operative samples and fill in a baseline questionnaire. They can donate a range of tissue samples according to their condition and treatment mode (e.g. fibroids or endometriotic tissue, peritoneal fluid), and are asked to repeatedly fill in follow-up questionnaires on their condition and quality of life, so that the clinical findings can be correlated with the outcome years later.

BMJ Open

A protocol for a longitudinal, prospective cohort study investigating the biology of uterine fibroids and endometriosis, and patients' quality of life – the FENOX study.

Journal:	*BMJ Open*
Manuscript ID	bmjopen-2019-032220.R2
Article Type:	Protocol
Date Submitted by the Author:	27-Jan-2020
Complete List of Authors:	Tapmeier, Thomas; University of Oxford, Nuffield Department of Women's & Reproductive Health<br>Nazri, Hannah; University of Oxford, Nuffield Department of Women's & Reproductive Health<br>Subramaniam, Kavita; University of Oxford, Nuffield Department of Women's & Reproductive Health<br>Manek, Sanjiv; Oxford University Hospitals NHS Foundation Trust, Department of Cellular Pathology<br>Garbutt, Kurtis; University of Oxford, Nuffield Department of Women's & Reproductive Health<br>Flint, Emma; University of Oxford, Nuffield Department of Women's & Reproductive Health<br>Cheuk, Cecilia; University of Oxford, Nuffield Department of Women's & Reproductive Health<br>Hubbard, Carol; University of Oxford, Nuffield Department of Women's & Reproductive Health<br>Barrett, Kelly; University of Oxford, Nuffield Department of Women's & Reproductive Health<br>Shepherd, Emily; University of Oxford, Nuffield Department of Women's & Reproductive Health<br>Zondervan, Krina; University of Oxford, Nuffield Department of Women's & Reproductive Health; Wellcome Trust Centre for Human Genetics<br>Becker, Christian; University of Oxford, Nuffield Department of Women's & Reproductive Health
**Primary Subject Heading**:	Obstetrics and gynaecology
Secondary Subject Heading:	Genetics and genomics, Reproductive medicine, Epidemiology
Keywords:	GYNAECOLOGY, Reproductive medicine < GYNAECOLOGY, Subfertility < GYNAECOLOGY, REPRODUCTIVE MEDICINE, SURGERY

SCHOLARONE™
Manuscripts

I, the Submitting Author has the right to grant and does grant on behalf of all authors of the Work (as defined in the below author licence), an exclusive licence and/or a non-exclusive licence for contributions from authors who are: i) UK Crown employees; ii) where BMJ has agreed a CC-BY licence shall apply, and/or iii) in accordance with the terms applicable for US Federal Government officers or employees acting as part of their official duties; on a worldwide, perpetual, irrevocable, royalty-free basis to BMJ Publishing Group Ltd ("BMJ") its licensees and where the relevant Journal is co-owned by BMJ to the co-owners of the Journal, to publish the Work in this journal and any other BMJ products and to exploit all rights, as set out in our licence.

The Submitting Author accepts and understands that any supply made under these terms is made by BMJ to the Submitting Author unless you are acting as an employee on behalf of your employer or a postgraduate student of an affiliated institution which is paying any applicable article publishing charge ("APC") for Open Access articles. Where the Submitting Author wishes to make the Work available on an Open Access basis (and intends to pay the relevant APC), the terms of reuse of such Open Access shall be governed by a Creative Commons licence – details of these licences and which Creative Commons licence will apply to this Work are set out in our licence referred to above.

Other than as permitted in any relevant BMJ Author's Self Archiving Policies, I confirm this Work has not been accepted for publication elsewhere, is not being considered for publication elsewhere and does not duplicate material already published. I confirm all authors consent to publication of this Work and authorise the granting of this licence.

For peer review only

Journal: **BMJ Open – Study Protocol**

Title: **A protocol for a longitudinal, prospective cohort study investigating the biology of uterine fibroids and endometriosis, and patients' quality of life – the FENOX study.**

Authors: Tapmeier TT[1], Nazri HM[1], Subramaniam KS[1], Manek S[2], Garbutt K[1], Flint EJ[1], Cheuk C[1], Hubbard C[1], Barrett K[1], Shepherd E[1], Zondervan KT[1,3], Becker CM[1]

[1]Endometriosis CaRe Centre, Nuffield Department of Women's & Reproductive Health, University of Oxford, Oxford, UK

[2]Department of Cellular Pathology, Oxford University Hospitals, Oxford, UK

[3]Wellcome Centre for Human Genetics, University of Oxford, Oxford

Corresponding authors: thomas.tapmeier@wrh.ox.ac.uk; christian.becker@wrh.ox.ac.uk

For peer review only

ABSTRACT

Introduction

Millions of women suffer from the consequences of endometriosis and uterine fibroids, with fibroids the cause for over 50% of hysterectomies in the US, and direct costs for their treatment estimated at between 4 and 9 billion USD. Endometriosis commonly affects millions of women worldwide predominantly during reproductive age, with severe menstrual and non-menstrual pain and subfertility the main symptoms. Due to the 'unhappy triad' of endometriosis – lack of awareness, lack of clinically relevant biomarkers and the unspecific nature of symptoms – women wait on average for 8–12 years before the definitive endometriosis diagnosis is made. Treatment options for both conditions are not satisfactory at the moment, especially with a view to preserving fertility for the women and families affected. In the Fibroids and Endometriosis Oxford (FENOX) study, we combine the investigation of fibroids and endometriosis, and plan to collect high quality tissue samples and medical data of participants over a time frame of 5 years after surgical intervention.

Methods and analysis

Biological samples such as blood, saliva, urine, fat, peritoneal fluid and – if found – endometrial tissue or fibroids as well as detailed clinical and intraoperative data will be collected from women undergoing surgery and participating in the study after informed consent. We plan to recruit up to 1200 participants per disease arm (i.e. endometriosis and uterine fibroids) over 5 years. Participants will fill in detailed and validated questionnaires on their medical history and quality of life, with follow-ups for 5 years. Enrolment started on 2nd April 2018, and FENOX will close on 31st March 2028. We will analyse the biological samples using state-of-the-art molecular biology methods and correlate the findings with the medical records and questionnaire data.

Ethics and dissemination

Approval for this study has been granted by the South Central - Oxford B Research Ethics Committee (REC No. 17/SC/0664), by the Health Research Authority (HRA), and by the Oxford University Hospitals NHS Foundation Trust. The findings will be published in high-ranking journals in the field and presented at national and international conferences.

Trial registration number

ISRCTN13560263; https://doi.org/10.1186/ISRCTN13560263

For peer review only

For peer review only - http://bmjopen.bmj.com/site/about/guidelines.xhtml

ARTICLE SUMMARY

Strengths and limitations of this study

- FENOX combines the study of endometriosis and uterine fibroids to identify the underlying mechanisms of both conditions.
- The study comprises biological samples as well as comprehensive phenotypic data.
- World Endometriosis Research Foundation (WERF) Endometriosis Phenome and Biobanking Harmonisation Project (EPHect) criteria are applied to ensure the best possible standardisation.
- The longitudinal aspect is an important feature of FENOX but this depends on uptake and compliance by participants.
- The control group comprises of women undergoing surgery for gynaecological indications other than endometriosis or uterine fibroids; thus, they are not completely healthy controls.

INTRODUCTION

Millions of women suffer from the consequences of endometriosis [1,2] and uterine fibroids [3,4]. These include pelvic and abdominal pain, abnormal uterine bleeding, infertility and miscarriages [5–8]. As such, these conditions not only affect women and their families in their everyday lives, but also have been shown to have an enormous socioeconomic impact for society in general: In the United States, fibroids are cited to be the cause for over 50% of hysterectomies [9], and direct costs for their treatment is estimated between 4 and 9 billion USD [10].

Clinically relevant, non-invasive diagnostic tests including biomarkers or imaging techniques do not exist for many forms of endometriosis [11–13] resulting in an average delay in diagnosis of 8-12 years. Current treatment options are associated with significant side effects and risks and include hormonal suppression/modification, surgical removal or, in the case of fibroids, embolization and MRI guided focussed ultrasound (MRgFUS).

Therefore, there exists a significant unmet clinical need to better understand the underlying mechanisms of these conditions, which will enable us to develop more specific diagnostic tests and will eventually lead to individualised treatment, with fewer side effects and better efficacy. To achieve this goal, it is essential to collect prospective high quality, standardised clinical and intra-operative data and corresponding biological samples. Our group has been at the forefront of the development of standard operating procedures and questionnaires for endometriosis as part of the World Endometriosis Research Foundation's (WERF) Endometriosis Phenome and Biobanking Harmonisation Project (EPHect) [14–17], and we are planning to establish similar standards in uterine fibroid research.

In the FENOX study, we aim to improve our understanding of the underlying mechanisms of endometriosis and uterine fibroids and their associated symptoms by means of longitudinal observation and laboratory analyses. To achieve this, samples and clinical data will be collected from

For peer review only - http://bmjopen.bmj.com/site/about/guidelines.xhtml

women undergoing surgery. These samples will be used in state-of-the-art biomedical assays (see 'Assays') to improve our understanding of the underlying biology of these symptoms in women with endometriosis and/or fibroids, which will lead to a better understanding of the conditions, stratification of patient groups and tailored therapies, and the development of novel drug targets and biomarkers for diagnosis and treatment.

Objectives

Primary objective

- To identify the underlying mechanisms of endometriosis and uterine fibroids and their associated symptoms to improve the outcome of affected women.

Secondary objectives

- To identify novel biomarkers of endometriosis.
- To identify clinical subgroups of endometriosis and uterine fibroids.
- To understand the genetics underlying these conditions and explore the relevant downstream molecular pathways.
- To investigate the relation between the presence of fibroids and the symptoms, e.g. abnormal uterine bleeding.
- To identify novel drug targets.
- To develop models of disease progression and prediction.
- To investigate conditions or symptoms associated with endometriosis and/or uterine fibroids, including: symptoms and characteristics of the female reproductive system (characteristics of menstrual bleeding, fertility, infertility, pregnancy outcomes), pelvic as well as non-pelvic pain conditions, metabolic phenotypes (polycystic ovarian syndrome (PCOS), obesity and fat distribution), cardiovascular conditions and symptoms, neuroangiogenesis and related neurological symptoms, immunological disorders, and cancers.

Outcomes

Primary outcome

We will used questionnaire data, medical records and sample analysis to investigate the genetic and molecular basis of the pathogenesis and symptoms of endometriosis and uterine fibroids. At the end of the recruiting period, i.e. from December 2022 onwards, the collected data and samples will be analysed and compared between endometriosis/fibroid cases, and non-affected controls.

For peer review only

For peer review only - http://bmjopen.bmj.com/site/about/guidelines.xhtml

Secondary outcomes

Prospective standardised questionnaires and samples will be collected according to EPHect standards. The correlation of cellular, molecular and genetic data and endometriosis status will allow us to define novel biomarkers of the disease.

Clinical notes and questionnaires in combination with sample data will be used to define clinical subgroups of patients.

The molecular and genetic findings will be compared against public databases of disease-relevant molecular pathways, and *in vitro* experiments will be carried out to test hypothetical connections between the genetics and manifestation of disease.

The blood vessels and endothelial cells will be compared between tissue from women presenting with fibroids and those without.

The detailed comparison between tissue from women with fibroids and those without will yield differences in terms of proteins expressed; these can then be tested as targets using known or new drugs.

As data accumulate and genetic mechanisms become clear, hypotheses will be formed as to the likely progression of disease. These will be tested against the reports from the follow-up questionnaires.

We will use questionnaire data, medical records and sample analysis to investigate the genetic and molecular basis of the pathogenesis and symptoms of conditions or symptoms associated with endometriosis and/or uterine fibroids.

METHODS AND ANALYSIS

Study design

FENOX is a prospective study that aims to improve our understanding of the underlying mechanisms of endometriosis and uterine fibroids and their associated symptoms by means of longitudinal observation and laboratory analyses. Biological samples such as blood, saliva, urine, fat, peritoneal fluid and – if found - endometriosis tissue or fibroids as well as detailed clinical and intraoperative data will be collected from women of reproductive age with and without endometriosis- and fibroid-associated symptoms, such as pain, abnormal uterine bleeding and infertility. Women undergoing surgery for these conditions, and women undergoing surgery for unrelated gynaecological conditions as part of their normal clinical management will be asked to participate (Fig. 1). An incidental diagnosis of endometriosis or uterine fibroids will lead to the patients' inclusion into the relevant case groups. All women attending clinics receive a letter informing them of ongoing research, and eligible women will be identified initially by research nurses or clinical staff during clinic visits. Once a woman has expressed an interest in participating in this study, they will be consented by a member of the research team (flowchart, Fig. 2).

Blood, saliva and urine will be taken prior to surgery. Tissue and peritoneal fluid (where applicable) will be taken at the time of the scheduled surgery.

For peer review only - http://bmjopen.bmj.com/site/about/guidelines.xhtml

In order to determine the effect of the surgical removal of the fibroids on the local tissue, it is necessary to take an additional endometrial biopsy after the planned surgical intervention. This sample will be timed to synch with the same time point in the menstrual cycle that the original sample was taken, and thus will give us a unique insight into the biology of the conditions. During this visit, blood and urine samples will be taken again also. Women can opt in or out of the additional clinic visit where these samples would be taken.

Women will be asked to complete questionnaires on paper, online or into their electronic handheld devices (health, pain, medication and, initially, ethnicity) at different time points. There will be a lengthy questionnaire at baseline before surgery (taking an estimated 45 minutes to complete), and shorter versions (taking up to 30 minutes to complete) post-operatively at 6-8 weeks, 6 months, 12 months and thereafter yearly for a total of five years after surgical intervention.

Samples

A. Blood samples (up to 50 ml, venepuncture), urine (micturition) and saliva (spit) will be taken prior to surgery.
B. During surgery, tissue samples will be taken as specified below.
C. In women opting in, an additional endometrial biopsy will be taken during a follow-up visit at least three months after surgery. This can be done in an outpatient setting, and the taking of an endometrial biopsy in this setting using an endometrial sampling device (e.g. pipelle or curette) is an established technique. A blood and urine sample will be taken again also.

Participants

In each of the disease arms (endometriosis or fibroids), we plan to recruit up to 1200 women of reproductive age (18 years until menopause) who are planned to undergo surgery. 800 of these will have the condition of interest, and 400 will be having surgery for other reasons and act as controls. In addition, we will include fibroid and uterine tissue samples collected as excess tissue (Oxford Radcliffe Biobank, REC Ref 09/H0606/5+5), currently approximately 70 samples.

Inclusion criteria

- The participant is willing and able to give informed consent for participation in the study.
- The participant is female and aged 18 years or above (before menopause).
- Women undergoing planned surgery (including hysterectomy) for endometriosis- and/or fibroid associated symptoms such as abdominal pain, abnormal uterine bleeding, or for unrelated gynaecological conditions (e.g. fertility investigation or for laparoscopic tubal sterilisation).

Exclusion criteria

The participant may not enter the study if ANY of the following apply:

For peer review only - http://bmjopen.bmj.com/site/about/guidelines.xhtml

- Women who are pregnant.
- Women who are unable to read, or to understand written or spoken English.
- History of cancer/ diagnosis of current cancer.

Participant enrolment

Recruitment

After general information through a generic letter with information about ongoing research, which every patient will receive prior to her outpatient appointment, eligible women will be identified initially by the research nurses or clinical team during clinic visits. The study research nurses will then contact those women interested in participating in the study.

Screening and Eligibility Assessment

Women attending clinic appointments for endometriosis- and fibroid-associated symptoms such as pain, abnormal uterine bleeding, and infertility will be asked to participate by clinical staff or by the authorised study research nurses. Women undergoing surgery for these conditions, and women undergoing surgery as part of their normal clinical management (e.g. laparoscopic tubal ligation or hysterectomy; they would be the control patients) are eligible to participate in the study.

Informed Consent

Prior to giving consent, and usually during their pre-operative assessment visit, women will be given the relevant patient information sheet and consent form to read. Written consent will be received by a trained member of the research team.

Written versions, with verbal explanations, of the patient information sheet and the consent forms will be presented to the participants detailing the exact nature of the study; what it will involve for the participant; the implications and constraints of the protocol; the known side effects and any risks involved in taking part. It will be clearly stated that the participant is free to withdraw from the study at any time for any reason without prejudice to future care, without affecting their legal rights, and with no obligation to give the reason for withdrawal.

The participant will be allowed as much time as wished to consider the information, and the opportunity to question the Investigator, their GP or other independent parties to decide whether they will participate in the study.

Written informed consent will then be obtained by means of the participant's dated signature and the dated signature of the person who presented and obtained the informed consent. The person who obtained the consent will be suitably qualified and experienced, and will have been authorised to do so by the Chief Investigator. A copy of the signed informed consent and the patient information sheet will be given to the participant. The original signed form will be retained at the study site. The consent form for this study allows for the participant declining consent for any procedure that she is not comfortable with, while remaining eligible as a participant of the study. E.g. if a participant did not want

For peer review only - http://bmjopen.bmj.com/site/about/guidelines.xhtml

a uterine biopsy used in the study, she would not initial the corresponding box on the consent form and insert ‘No’ instead.

Study settings

Baseline Assessments

Consented participants will be asked by the research team to complete a baseline questionnaire before their surgery. This may be sent to them e.g. via mail or e-mail before surgery or given to them in paper form in the clinic. Alternatively, participants have the option to complete an online version of the questionnaire or they may fill in the questionnaire on their hand-held device. Each participant will receive a unique login for the online questionnaires.

The questionnaire data will be withheld from the research team until written informed consent is obtained and will be destroyed if this is not granted.

All participants will be sent further questionnaires at different time points (approximately 6-8 weeks, 6 months, 12 months and then yearly for 5 years after surgery). Participants may be reminded (twice, maximally) to return completed questionnaires via mail, email, phone or text or similar.

On the day of surgery, they will be asked to provide a mid-stream urine and a saliva sample. In addition, blood will be collected by peripheral venepuncture. The procedures will be explained to the women again and they will be given the opportunity to ask questions. Assessment of the presence and extent of disease will be performed by the operating surgeon.

Samples that may be taken at time of surgical procedures are as follows:

1. *Laparoscopy for suspected endometriosis:*

Endometrial biopsy, peritoneal fluid aspiration, fat tissue biopsy, endometriotic tissue if present, peritoneal biopsy.

2. *Laparoscopy for uterine fibroids:*

Endometrial biopsy, peritoneal fluid aspiration, fat tissue biopsy, endometriotic tissue (if present), peritoneal biopsy, fibroid tissue, myometrial biopsy.

3. *Laparotomy for uterine fibroids:*

Endometrial biopsy, peritoneal fluid aspiration, fat tissue biopsy, endometriotic tissue if present, peritoneal biopsy, fibroid tissue, myometrial biopsy.

4. *Trans-cervical resection of uterine fibroids (TCRF):*

Endometrial biopsy, fibroid tissue, myometrial biopsy

For peer review only - http://bmjopen.bmj.com/site/about/guidelines.xhtml

For peer review only

5. *Laparoscopy for tubal sterilisation (Controls):*

Endometrial biopsy, peritoneal fluid aspiration, fat tissue biopsy, peritoneal biopsy, myometrial biopsy

6. *Hysterectomy:*

Whole uterus as excess tissue from women with fibroids as well as from controls without fibroids, who undergo surgery for other indications (such as heavy menstrual bleeding, or pain), peritoneal fluid aspiration, fat tissue biopsy and peritoneal biopsy.

Women undergoing hysterectomy for benign causes such as abnormal uterine bleeding or pain will be asked to donate part of their uterus for research. Hysterectomy specimens are excess tissue and would be discarded otherwise.

During surgery, the surgeon will record digital photographs of the inside of the abdomen and/or uterine cavity as part of routine clinical care, which will also be stored on a secure server identified by the participant's study ID. Intraoperative findings will be recorded by the surgeon and anonymised data collected.

Subsequent Visits

Unless participants underwent a hysterectomy, all included women will be asked to contact the study team when the next menstrual period after the procedure started. Together with the last menstrual period (LMP) date given at the time of the procedure, this date will be used to calculate the length of the cycle. This is important in order to account for the changes that occur in the uterus during the menstrual cycle, and to enable us to distinguish between the effects of the cycle and the disease.

One subsequent visit will be made by participants treated for fibroids who consent to this. They will have another endometrial sample taken at least three months after the surgical intervention in an outpatient setting. The taking of an endometrial biopsy in this setting using an endometrial sampling device (e.g. pipelle or curette) is an established technique, takes approximately 30 minutes, and there is only a minimal risk of bleeding. In addition, blood and urine samples will be taken also. Pregnant women will not be eligible for the subsequent visits.

All women will be contacted by a member of the medical or study team and asked to fill in further questionnaires at different follow-up time points (approximately 6-8 weeks, 6 months, 12 months and yearly thereafter for a total of 5 years). Reminders will be sent twice, maximally. Women will also be asked if they can be contacted in the future for any further studies approved by an ethics committee.

For peer review only - http://bmjopen.bmj.com/site/about/guidelines.xhtml

Sample Handling

Samples will be obtained according to WERF/EPHect guidelines[14–17] by the interventions listed below. Only the study team will have access. Biological samples will be stored at -20°C or at -80°C for use in current and future studies until exhausted. Disease and control samples will be stored under the same conditions. For the purposes of this study, samples may be analysed at Oxford, or they may be transferred to a third party/study collaborator, including industrial partners, for analysis at their facility. If participants agree, samples will be moved to a Research Tissue Bank at the end of the study or stored and used in future ethically approved studies. They would be made available in anonymised form.

To investigate the relationship between uterine fibroids and symptoms such as abnormal uterine bleeding and pain, we aim to collect endometrial and myometrial samples alongside the fibroids themselves, to be able to detect the effects of fibroids on their surroundings.

We also intend to use the endometrium and endometriotic samples, one of the blood samples and fat samples to look for genetic factors and molecular pathways that can lead to endometriosis or uterine fibroids. The samples for this analysis will also be anonymised so that we do not know specifically which patient they came from. However, all samples are identifiable with printed label and location detail, participant ID, sample type and colour-coded cap.

Blood:

50 mL. These are divided into (at least) EDTA- (2 x 9 mL) and heparin-treated samples (2 x 6 mL, both anti-coagulation), serum (2 x SST, 5 mL) and two plain blood samples of 5 mL. The different vials are colour-coded and frozen at -80°C.

Urine:

20 mL. Half of the sample will be used to test for glucose by specific gravity assay, the other half will be stored for the study. One aliquot of 5 mL is frozen directly at -80°C; 1 mL is centrifuged at 300 g, and 5 aliquots a 200 µL of cell-free supernatant are frozen at -80°C.

Saliva:

A spit sample of approximately 1 mL is taken on ice. One aliquot of 200 µL is frozen at -80°C, the rest is centrifuged at 300 g and 2 x 200 µL of cell-free supernatant are frozen at -80°C. One aliquot of 50 µL of cell-free supernatant is combined with 200 µL of RNA-preserving buffer (RNA later, Qiagen, Germany) and then frozen at -80°C.

Peritoneal fluid:

During surgery, the peritoneal fluid will be collected by the surgeon using a syringe or through mechanical suction on ice. Depending on the volume (up to 15 mL), an aliquot will be centrifuged at 300 g, and the pellet (cells) stored at -80°C for further analysis. The cell-free supernatant will also be stored at -80°C.

For peer review only - http://bmjopen.bmj.com/site/about/guidelines.xhtml

Endometrium (e.g. pipelle or curette), endometrial lesions (peritoneum), abdominal fat, myometrium, fibroid tissue:

All tissue will be collected on ice and divided for storage at -80°C and – after fixing in paraformaldehyde and ethanol – at room temperature. Parts of fresh tissues will be used for culturing experiments, in order to test compounds, drugs or similar agents on primary cells.

Hysterectomy:

In agreement with the local pathologist, whole uteri will be taken on ice and used for perfusion experiments within 24 hours before being transferred to pathology. Tissue samples of myometrium, endometrium, fibroid and fibroid-associated vasculature (if present) will be taken and stored at -80°C and – after fixing in paraformaldehyde and ethanol – at room temperature as the other tissue samples above.

Assays

RNA analysis

RNA from each sample will be isolated by standard methods. Gene expression studies will be carried out between cases and controls (e.g. endometriosis vs non-endometriosis patients, or fibroid bearing women vs women without fibroids) using quantitative real-time PCR assays, whole RNA sequencing methods and RNA microarrays.

Protein analysis

Proteins will be extracted from tissue samples using standard methods. The expression and amount of proteins will be analysed by immunoblotting for specific proteins of interest, and by proteomics methods using the matrix-assisted laser desorption/ionization (MALDI)/surface-enhanced laser desorption/ionization (SELDI) platform. Tissue sections will be used in standard immunohistochemistry to detect the expression of markers of interest *in situ*.

Cells

Fresh tissue will be dissociated into single cell suspensions. From these, the diverse cell types (e.g. endothelial cells) will be grown in incubators *in vitro* in order to study differences in cell behaviour between cases and controls, and to test compounds and drugs. Cells will be analysed by microscopy, flow cytometry and immunocytochemistry methods.

Similarly, cells isolated from peritoneal fluid or blood will be analysed using these methods.

Secretome analysis (perfusion)

Whole uteri with and without fibroids will be perfused with a suitable buffer for up to 8 hours. The perfusate will be analysed by proteomics methods (see above) to detect factors secreted by the fibroids.

For peer review only

For peer review only - http://bmjopen.bmj.com/site/about/guidelines.xhtml

Microscopy

Tissue blocks (up to 5 cubic millimetres in size) from perfused uteri will be stained with antibodies against markers of blood vessels and fibroids, and leakiness, and be recorded in a confocal microscope in order to render a three-dimensional image of the blood vessels *in situ*. The detailed study of these will allow us to determine whether there is a significant difference in the architecture of blood vessels in uteri with fibroids compared to those from uteri without fibroids.

Discontinuation/Withdrawal of Participants from Study

Each participant has the right to withdraw from the study at any time. In addition, the Investigator may discontinue a participant from the study at any time if the Investigator considers it necessary for any reason including:

- Pregnancy
- Ineligibility (either arising during the study or retrospectively, having been missed at screening)
- Withdrawal of Consent
- Loss to follow up
- Loss of mental capacity

Withdrawal from the study: At the point the participant withdraws from the study, we will ask for consent to retain samples and data collected up to that point. Withdrawn participants will not be replaced. The reason for withdrawal will be recorded in the case record file (CRF).

Definition of End of Study

The end of study is six months after the locking of the study database, to allow for completion of data analysis.

Patient and Public Involvement

FENOX was built on experience and feedback we received from patients and research nurses during a previous study (A study to identify possible biomarkers in women with Endometriosis at Oxford – ENDOX[18]). In addition, the research objectives were set in accordance with research priorities identified through the James Lind Alliance Priority Setting Partnership (PSP) for endometriosis, in which we participate[19]. The James Lind Alliance brings patients, carers and clinicians together in Priority Setting Partnerships (PSPs) to identify and prioritise the Top 10 unanswered questions or evidence uncertainties that they agree are the most important.

INTERVENTIONS

Non-clinical Interventions

Questionnaires:

Participants will complete specific questionnaires about their condition, general health, pain sensitivity, medication and menstrual history before their surgery. Additionally, those women with endometriosis

For peer review only - http://bmjopen.bmj.com/site/about/guidelines.xhtml

will be asked to complete the Endometriosis Health Profile (EHP-30) Questionnaire[20], while women with fibroids complete a section on their quality of life (UFS-QoL[21]). Currently, the clinical questionnaires are completed by participants on paper; in the future, the aim is that the questionnaires can be completed via a handheld device or via the internet directly onto a secure server. For this, women will receive a pre-trial study number and login information. The follow-up questionnaires ask about symptoms and changes in menstrual history as relevant. The control groups would be given the same questionnaires as the women with the respective condition and asked to omit questions not applicable to them.

Medical records:

We will obtain clinical data (menstrual cycle phase, medication, pain and menstrual bleeding status, photos from surgery) from the patients' medical records.

Clinical Interventions

Venepuncture:

Taken by an appropriately trained member of the clinical or research staff.

Collection of other bodily fluid sample:

Urine and saliva samples donated by the patient and sample prepared and analysed by a member of the investigative team.

Tissue collection:

Tissue/fluid (e.g. fibroids if present) will be collected as part of routine surgical management apart from:

Laparoscopy:

Peritoneal fluid will be aspirated, biopsies from endometrium (e.g. pipelle or curette), abdominal fat tissue, myometrium, and peritoneum (excision) will be taken during surgery.

Additional Risk:

Minor bleeding, uterine perforation (<1%), additional length of surgery by 5 min.

Myomectomy/hysterectomy:

Endometrial, myometrial and/or fibroid tissue biopsies will be taken during surgery. Hysterectomy samples will be used in structural analysis assays *ex vivo* in close discussion with the clinical pathologists.

Additional Risk: Minor bleeding, uterine perforation (<1%), additional length of surgery by 5 min.

Trans-cervical resection of fibroids:

Endometrial and myometrial biopsies will be taken during surgery.

Additional Risk: Minor bleeding, uterine perforation (<1%), additional length of surgery by 5 min.

For peer review only - http://bmjopen.bmj.com/site/about/guidelines.xhtml

Additionally:

In women opting in, an additional endometrial biopsy will be taken during a follow-up visit n an outpatient setting. The biopsy of the endometrium is a simple, routine procedure and takes about 30 minutes.

Additional risk: Minor bleeding, uterine perforation (<1%), short period of discomfort.

Women will be asked to consent to the use of samples and clinical data collected as part of this research and in future research. Women, if they consent, will potentially be contacted for future studies approved by an ethics committee.

Adverse events

For this study, it is conceivable that additional procedures may result in bleeding. However, if this resulted in a scenario mentioned below, it would constitute an SAE and needed to be reported to the sponsor.

A serious adverse event is any untoward medical occurrence that:

- results in death
- is life-threatening
- requires inpatient hospitalisation or prolongation of existing hospitalisation
- results in persistent or significant disability/incapacity
- consists of a congenital anomaly or birth defect

Other 'important medical events' may also be considered serious if they jeopardise the participant or require an intervention to prevent one of the above consequences.

NOTE: The term "life-threatening" in the definition of "serious" refers to an event in which the participant was at risk of death at the time of the event; it does not refer to an event which hypothetically might have caused death if it were more severe.

A serious adverse event (SAE) occurring to a participant should be reported to the REC that gave a favourable opinion of the study where in the opinion of the Chief Investigator the event was 'related' (resulted from administration of any of the research procedures) and 'unexpected' in relation to those procedures. Reports of related and unexpected SAEs should be submitted within 15 working days of the Chief Investigator becoming aware of the event, using the HRA report of serious adverse event form (see HRA website).

Data Analysis Plan

As this is a prospective sample and data collection study, there is no randomisation of patients as all women will undergo surgery as part of their routine clinical management. As previously[18], we will use SPSS, Graph Pad Prism, STATA and R for analysis, and employ t-tests, ANOVA, correlation coefficient

For peer review only - http://bmjopen.bmj.com/site/about/guidelines.xhtml

analysis and similar methods. We plan to use multivariate logistic regression models in comparisons of endometriosis cases with controls, and fibroid cases with controls, adjusting for confounders relevant to the hypothesis being tested. A priori confounders are likely to be age, ethnicity and menstrual cycle phase. Patients with both endometriosis and fibroids will enter into the analysis according to the research question asked; we will conduct sensitivity analyses on this comorbid group to examine to what extent they influence the results. Due to the exploratory nature of this study, various additional statistical techniques may also be used to fully explore the relationships in the data, but all methods will be fully documented.

Power Calculations

Power calculations were done in R (v3.6.1) using the ***pwr*** package. For our primary outcome we plan to correlate questionnaire and laboratory data. To detect correlations with a moderate effect size of r=0.3 at a power of 80% and 0.05 significance level, we will need 85 samples per group (***pwr.r.test***). For the detection of effect sizes of at least 0.2 between groups (e.g. endometriosis cases vs controls, with 3 cycle phases and 5 disease stages (0, stages 1 – 4) using ANOVA at 0.05 significance and 80% power, at least 32 samples per groups will be used, with a total of 480 samples for all 15 groups (***pwr.anova.test***). Multiple comparisons will be corrected for by Bonferroni's method.

The Number of Participants

It is now recognised that both endometriosis and uterine fibroids are very heterogeneous conditions. Our previous studies[22,23] and systematic reviews[24,25] have clearly identified a lack of sufficiently powered studies. Multiple large-scale research collaborations are currently in place investigating different aspects of endometriosis[26], and we plan similar efforts for uterine fibroids. Therefore, large patient numbers are needed.

The Endometriosis CaRe Centre at Oxford is the UK's largest endometriosis centre. Similarly, as a tertiary referral centre, we see many women with fibroid–associated symptoms. As a result, we have the unique opportunity to collect large amounts of data and samples, which is essential to produce clinically meaningful outputs. Given our current patient recruitment rate (endometriosis: 100/year, uterine fibroids, 200/year) we estimate an enrolment of approximately 2 × 1200 women over the course of the study (800 endometriosis patients + 400 non-endometriotic controls, 800 fibroid patients + 400 non-fibroid controls). Fibroids already collected as excess tissue under the Oxford Radcliffe Biobank (ORB, REC Ref 09/H0606/5+5) will also be included in this study, currently approximately 70 samples.

Analysis of Outcome Measures

All samples excluding those from patients who withdraw consent will be included in the analysis of outcome measures.

Laboratory data will be analysed using assay-specific software packages employing univariate and multivariate pattern recognition methods (e.g. principal component analysis, partial least squares, stochastic neighbour embedding algorithms) between sample groups. Correlation with questionnaire data will allow us to validate prospective markers of disease. In addition, we will use laboratory data to

For peer review only - http://bmjopen.bmj.com/site/about/guidelines.xhtml

predict disease severity (revised American Fertility Society score[27]), quality of life (EHP-30[20]), pain measures and improvement of symptoms as per follow-up questionnaires. For the multivariate predictive methods, a test set of approximately 30% of each treatment group will be selected at random. This may be selected in a stratified method and exclude patients that have particularly extreme values (e.g. > 3 SD from the mean). Patients not included in the test set will make up the training set. Models will then be built on the training set and assessed for predictability on the test set. The final analysis will be performed on the whole data set. However, if some influential differences e.g. in BMI or comorbidities are seen, then e.g. the women with endometriosis will be matched to corresponding women without endometriosis, or women with fibroids to women without fibroids, and the analysis based on these matched pairs.

DATA MANAGEMENT

Access to Data

Direct access will be granted to authorised representatives from the Sponsor and host institution for monitoring and/or audit of the study to ensure compliance with regulations.

Data Recording and Record Keeping

Each participant will receive a unique study number, which will then be used throughout the study. A study master sheet linking patient identifiable data (name, DOB, hospital and NHS numbers) with the unique study number will be kept and password protected on the University of Oxford's High Compliance server with authorised access and in a file separate from the main study file. Hard copy study documents will be kept in a locked room at each participating centre. Research data will therefore be using non-identifiable data, and all records will be identified only by this study number. All study data will be entered on a desktop computer into a program such as Microsoft EXCEL or Sapphire (Labvantage) using password protection. The participants will be identified by study number in any database. The name and any other identifying details will NOT be included in any electronic file of study data.

Where participants consent, coded genetic data and limited relevant details including, age, gender, information about body habitus, biochemistry etc. can also be made available to collaborators and to the National Institute for Health Research (NIHR) Bioresource (http://bioresource.nihr.ac.uk/), a panel of thousands of volunteers, who are willing to be approached to participate in research studies investigating the links between genes, the environment, health and disease.

QUALITY ASSURANCE PROCEDURES

The study may be monitored, or audited in accordance with the current approved protocol, GCP, relevant regulations and standard operating procedures.

For peer review only - http://bmjopen.bmj.com/site/about/guidelines.xhtml

541 **ETHICS AND DISSEMINATION**

542 **Declaration of Helsinki**

543 This study will be conducted in accordance with the principles of the Declaration of Helsinki.

544 **Guidelines for Good Clinical Practice**

545 The Investigator will ensure that this study is conducted in accordance with relevant regulations and
546 with Good Clinical Practice.

547 **Approvals**

548 Approval for this study has been granted by the South Central - Oxford B Research Ethics Committee
549 (REC No. 17/SC/0664) on 31st January 2018, by the Health Research Authority (HRA) on 28th February
550 2018, and by the Oxford University Hospitals NHS Foundation Trust on 20th March 2018.

551 **Reporting**

552 The CI shall submit once a year throughout the study, or on request, an Annual Progress report to the
553 REC Committee, HRA (where required) host organisation and Sponsor. In addition, an End of Study
554 notification and final report will be submitted to the same parties.

555 **Publications**

556 The findings from the study will be published in high-ranking journals in the field and presented at
557 national and international conferences.

558 **Participant Confidentiality**

559 The study staff will ensure that the participants' anonymity is maintained. The participants will be
560 identified only by a participant ID number on all study documents and any electronic database, with
561 the exception of the CRF, where participant initials may be added. All documents will be stored securely
562 and only accessible by study staff and authorised personnel. The study will comply with the Data
563 Protection Act, which requires data to be anonymised as soon as it is practical to do so.

564 **Expenses and Benefits**

565 There will be no payments made to study participants.

566 **Other Ethical Considerations**

567 Participants unable to consent for themselves will not be included in the study.

568 Patients under clinical management for infertility will be approached in a most sensitive manner by our
569 experienced and well-trained team. It is unlikely that our genetic analysis of the participants will reveal
570 anything relevant beyond their normal clinical care so we do not plan to report any such findings to
571 them or their GPs.

572 **DISCUSSION**

573 This study has been designed to address both uterine fibroids and endometriosis as conditions that
574 both affect the female reproductive system and pose similar problems with regards to pain treatment,
575 fertility and quality of life. By combining the patient collectives into one study, we hope to make use of
576 synergies between the investigations of the two conditions, in addition to the apparent comorbidity
577 between endometriosis patients and those with uterine fibroids [28,29]. Uniquely, the study protocol

For peer review only - http://bmjopen.bmj.com/site/about/guidelines.xhtml

allows for the sampling of endometrium on a follow-up visit, which will allow for the assessment of the local, molecular effects of treatment within the same participant.

FENOX has been designed with the EPHect principles [14–17] in mind to ensure standardisation and reproducibility, and thus should deliver high-quality datasets that will be useful and comparable between centres. We are currently expanding the collection of samples to sites outside of Oxford, with a view to make FENOX a multi-centre study within the UK eventually.

Author affiliations

[1]Endometriosis CaRe Centre, Nuffield Department of Women's & Reproductive Health, University of Oxford, Oxford, UK

[2]Department of Cellular Pathology, Oxford University Hospitals, Oxford, UK

[3]Wellcome Centre for Human Genetics, University of Oxford, Oxford

Acknowledgements

We thank all women who participated in FENOX and its predecessor, ENDOX. We acknowledge the indispensable help and expertise of Fiona Goddard, NDWRH, and Karen Melham, CTRG, University of Oxford, in preparing the study protocol.

Contributors

TTT wrote the study protocol with KTZ and CMB. CH, KB and ES consented participants and collected samples. HMN, KSS and TTT processed samples. KG, EJF and CC documented samples and designed electronic questionnaires. SM determined menstrual cycle stages by histology. KTZ and CMB conceived the study.

Funding

TTT received funding from the Nuffield Benefaction for Medicine and the Wellcome Institutional Strategic Support Fund (ISSF, ref no. 5258). HMN received funding from the Oxfordshire Health Services Research Committee. KG was supported by a grant from the National Institutes of Health USA (R01HD094842). Funding for this study has been obtained from the Nuffield Department of Women's & Reproductive Health under the Oxford/Bayer-Alliance for Women's Health.

Competing interests

CMB and KTZ received research grants from Bayer Healthcare, Volition RX, MDNA Life Sciences and Roche Diagnostics. The study is funded by the Nuffield Department of Women's & Reproductive Health.

Patient consent

See above, *informed consent*

For peer review only

For peer review only - http://bmjopen.bmj.com/site/about/guidelines.xhtml

Ethics approval

Approval has been granted by the South Central - Oxford B Research Ethics Committee (REC No. 17/SC/0664), by the Health Research Authority (HRA), and by the Oxford University Hospitals NHS Foundation Trust.

Provenance and peer review

The FENOX protocol was based upon the previous ENDOX study (REC reference 09/H0604/58). It was originally approved in January 2018, with amendments approved in April 2019.

Open access

The study protocol will be published under a Creative Commons licence.

Data sharing

The authors will make relevant anonymised patient-level data available upon reasonable request, according to the established standards in the field. Data that could compromise participant anonymity or privacy will not be shared in any way.

For peer review only

For peer review only - http://bmjopen.bmj.com/site/about/guidelines.xhtml

REFERENCES

1 Berkley KJ. The Pains of Endometriosis. *Science (80-)* 2005;**308**:1587–9. doi:10.1126/science.1111445

2 Zondervan KT, Becker CM, Koga K, *et al.* Endometriosis. *Nat Rev Dis Prim* 2018;**4**:9. doi:10.1038/s41572-018-0008-5

3 Peddada SD, Laughlin SK, Miner K, *et al.* Growth of uterine leiomyomata among premenopausal black and white women. *Proc Natl Acad Sci* 2008;**105**:19887–92. doi:10.1073/pnas.0808188105

4 Buttram VC, Reiter RC. Uterine leiomyomata: etiology, symptomatology, and management. *Fertil Steril* 1981;**36**:433–45.http://www.ncbi.nlm.nih.gov/pubmed/7026295

5 Burney RO, Giudice LC. Pathogenesis and pathophysiology of endometriosis. *Fertil Steril* 2012;**98**:511–9. doi:10.1016/j.fertnstert.2012.06.029

6 Giudice LC. Clinical practice. Endometriosis. *N Engl J Med* 2010;**362**:2389–98. doi:10.1056/NEJMcp1000274

7 Gupta S, Jose J, Manyonda I. Clinical presentation of fibroids. *Best Pract Res Clin Obstet Gynaecol* 2008;**22**:615–26. doi:10.1016/j.bpobgyn.2008.01.008

8 Longo DL, Bulun SE. Uterine Fibroids. *N Engl J Med* 2013;**369**:1344–55. doi:10.1056/NEJMra1209993

9 Aarts JWM, Nieboer TE, Johnson N, *et al.* Surgical approach to hysterectomy for benign gynaecological disease. *Cochrane Database Syst Rev* Published Online First: 2015. doi:10.1002/14651858.CD003677.pub5

10 Cardozo ER, Clark AD, Banks NK, *et al.* The estimated annual cost of uterine leiomyomata in the United States. *Am J Obstet Gynecol* 2012;**206**:211.e1-211.e9. doi:10.1016/j.ajog.2011.12.002

11 Gupta D, Hull ML, Fraser I, *et al.* Endometrial biomarkers for the non-invasive diagnosis of endometriosis. *Cochrane database Syst Rev* 2016;**4**:CD012165. doi:10.1002/14651858.CD012165

12 Nisenblat V, Prentice L, Bossuyt PM, *et al.* Combination of the non-invasive tests for the diagnosis of endometriosis. *Cochrane Database Syst Rev* 2016;**7**:CD012281. doi:10.1002/14651858.CD012281

13 Nisenblat V, Bossuyt PM, Shaikh R, *et al.* Blood biomarkers for the non-invasive diagnosis of endometriosis. *Cochrane Database Syst Rev* 2016;:CD012179. doi:10.1002/14651858.CD012179

14 Becker CM, Laufer MR, Stratton P, *et al.* World Endometriosis Research Foundation Endometriosis Phenome and Biobanking Harmonisation Project: I. Surgical phenotype data collection in endometriosis research. *Fertil Steril* 2014;**102**:1213–22. doi:10.1016/j.fertnstert.2014.07.709

15 Vitonis AF, Vincent K, Rahmioglu N, *et al.* World Endometriosis Research Foundation Endometriosis Phenome and Biobanking Harmonization Project: II. Clinical and covariate

For peer review only - http://bmjopen.bmj.com/site/about/guidelines.xhtml

phenotype data collection in endometriosis research. *Fertil Steril* 2014;**102**:1223–32. doi:10.1016/j.fertnstert.2014.07.1244

16 Rahmioglu N, Fassbender A, Vitonis AF, *et al.* World Endometriosis Research Foundation Endometriosis Phenome and Biobanking Harmonization Project: III. Fluid biospecimen collection, processing, and storage in endometriosis research. *Fertil Steril* 2014;**102**:1233–43. doi:10.1016/j.fertnstert.2014.07.1208

17 Fassbender A, Rahmioglu N, Vitonis AF, *et al.* World Endometriosis Research Foundation Endometriosis Phenome and Biobanking Harmonisation Project: IV. Tissue collection, processing, and storage in endometriosis research. *Fertil Steril* 2014;**102**:1244–53. doi:10.1016/j.fertnstert.2014.07.1209

18 Rahmioglu N, Drong AW, Lockstone H, *et al.* Variability of genome-wide DNA methylation and mRNA expression profiles in reproductive and endocrine disease related tissues. *Epigenetics* 2017;**12**:897–908. doi:10.1080/15592294.2017.1367475

19 Horne AW, Saunders PTK, Abokhrais IM, *et al.* Top ten endometriosis research priorities in the UK and Ireland. *Lancet (London, England)* 2017;**389**:2191–2. doi:10.1016/S0140-6736(17)31344-2

20 Jones G. Development of an endometriosis quality-of-life instrument: The Endometriosis Health Profile-30. *Obstet Gynecol* 2001;**98**:258–64. doi:10.1016/S0029-7844(01)01433-8

21 Spies JB, Coyne K, Guaou Guaou N, *et al.* The UFS-QOL, a new disease-specific symptom and health-related quality of life questionnaire for leiomyomata. *Obstet Gynecol* 2002;**99**:290–300.http://www.ncbi.nlm.nih.gov/pubmed/11814511

22 Rahmioglu N, Nyholt DR, Morris AP, *et al.* Genetic variants underlying risk of endometriosis: insights from meta-analysis of eight genome-wide association and replication datasets. *Hum Reprod Update* 2014;**20**:702–16. doi:10.1093/humupd/dmu015

23 Fung JN, Rogers PAW, Montgomery GW. Identifying the Biological Basis of GWAS Hits for Endometriosis. *Biol Reprod* 2015;**92**:87–87. doi:10.1095/biolreprod.114.126458

24 May KE, Villar J, Kirtley S, *et al.* Endometrial alterations in endometriosis: a systematic review of putative biomarkers. *Hum Reprod Update* 2011;**17**:637–53. doi:10.1093/humupd/dmr013

25 May KE, Conduit-Hulbert SA, Villar J, *et al.* Peripheral biomarkers of endometriosis: a systematic review. *Hum Reprod Update* 2010;**16**:651–74. doi:10.1093/humupd/dmq009

26 Sapkota Y, Steinthorsdottir V, Morris AP, *et al.* Meta-analysis identifies five novel loci associated with endometriosis highlighting key genes involved in hormone metabolism. *Nat Commun* 2017;**8**:15539. doi:10.1038/ncomms15539

27 The American Society for Reproductive Medicine. Revised American Society for Reproductive Medicine classification of endometriosis: 1996. *Fertil Steril* 1997;**67**:817–21.http://www.ncbi.nlm.nih.gov/pubmed/9130884

28 Choi EJ, Cho SB, Lee SR, *et al.* Comorbidity of gynecological and non-gynecological diseases with adenomyosis and endometriosis. *Obstet Gynecol Sci* 2017;**60**:579–86.

For peer review only

For peer review only - http://bmjopen.bmj.com/site/about/guidelines.xhtml

doi:10.5468/ogs.2017.60.6.579

29 Huang JQ, Lathi RB, Lemyre M, *et al.* Coexistence of endometriosis in women with symptomatic leiomyomas. *Fertil Steril* 2010;**94**:720–3. doi:10.1016/j.fertnstert.2009.03.052

For peer review only

For peer review only - http://bmjopen.bmj.com/site/about/guidelines.xhtml

FIGURE LEGENDS

Figure 1: Participant groups and procedures. The participants will be allocated into case or control groups and tissues will be sourced according to the course of clinical intervention: Participants with fibroids treated by transcervical resection (TCRF) will not undergo abdominal surgery, while abdominal surgery is necessary if the fibroids are treated by laparoscopy, open myomectomy or hysterectomy. In these cases, peritoneal fluid can be obtained in addition to the tissue samples (fibroid, myometrium, endometrium). Endometriosis patients will undergo laparoscopy. If found without endometriosis, they will be grouped with the controls, who are women undergoing surgery for other, unrelated conditions (e.g. tubular ligation).

Figure 2: Flowchart of participant data and tissue sampling. Consented participants of the different arms of the study donate pre-operative samples and fill in a baseline questionnaire. They can donate a range of tissue samples according to their condition and treatment mode (e.g. fibroids or endometriotic tissue, peritoneal fluid), and are asked to repeatedly fill in follow-up questionnaires on their condition and quality of life, so that the clinical findings can be correlated with the outcome years later.

For peer review only - http://bmjopen.bmj.com/site/about/guidelines.xhtml

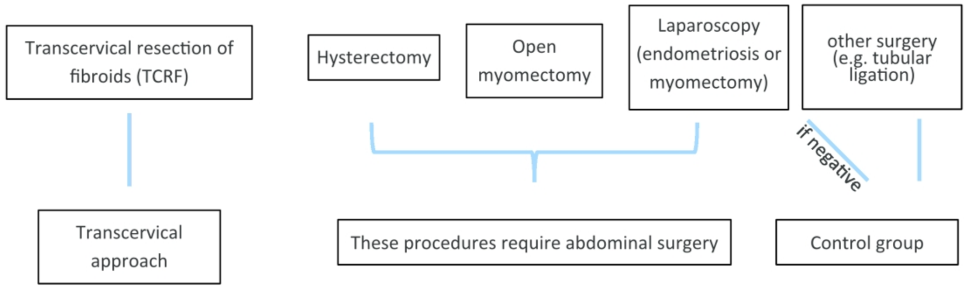

Figure 1: Participant groups and procedures. The participants will be allocated into case or control groups and tissues will be sourced according to the course of clinical intervention: Participants with fibroids treated by transcervical resection (TCRF) will not undergo abdominal surgery, while abdominal surgery is necessary if the fibroids are treated by laparoscopy, open myomectomy or hysterectomy. In these cases, peritoneal fluid can be obtained in addition to the tissue samples (fibroid, myometrium, endometrium). Endometriosis patients will undergo laparoscopy. If found without endometriosis, they will be grouped with the controls, who are women undergoing surgery for other, unrelated conditions (e.g. tubular ligation).

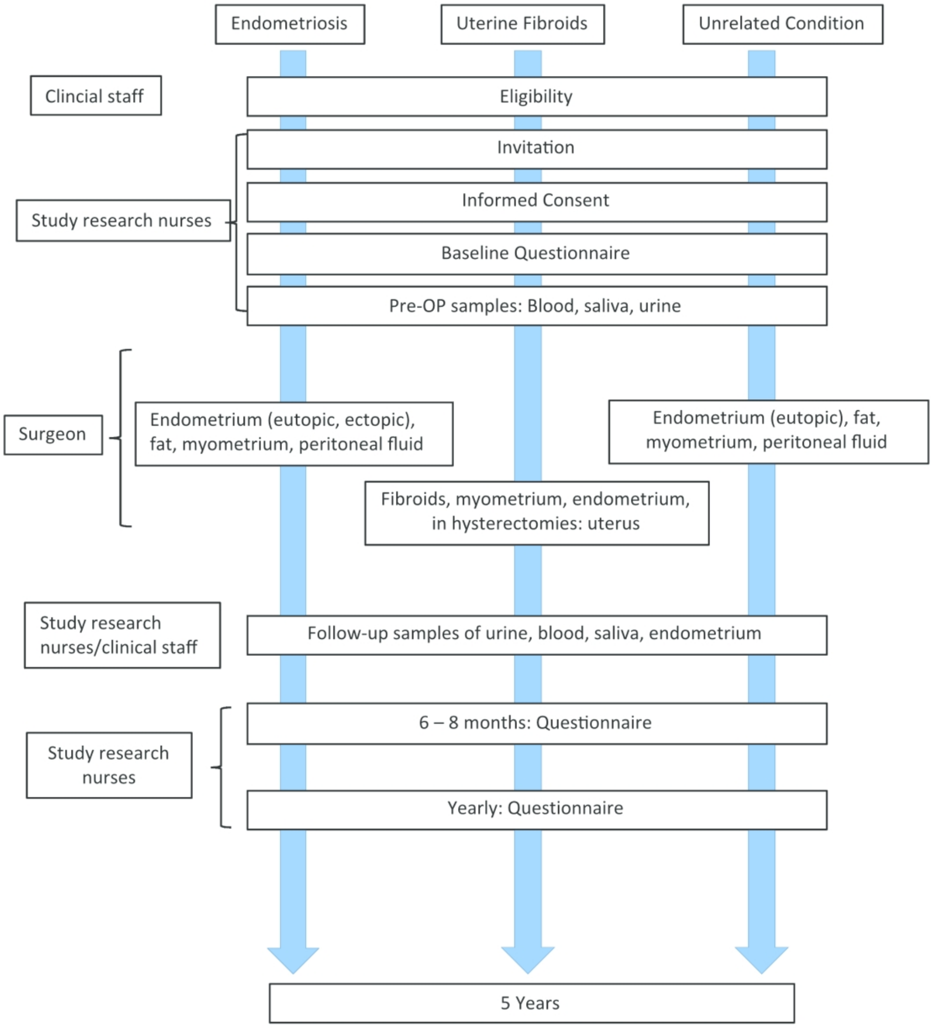

Figure 2: Flowchart of participant data and tissue sampling. Consented participants of the different arms of the study donate pre-operative samples and fill in a baseline questionnaire. They can donate a range of tissue samples according to their condition and treatment mode (e.g. fibroids or endometriotic tissue, peritoneal fluid), and are asked to repeatedly fill in follow-up questionnaires on their condition and quality of life, so that the clinical findings can be correlated with the outcome years later.
